# Tumor Suppressive Role of miR-342-5p in Human Chondrosarcoma Cells and 3D Organoids

**DOI:** 10.3390/ijms22115590

**Published:** 2021-05-25

**Authors:** Clément Veys, Abderrahim Benmoussa, Romain Contentin, Amandine Duchemin, Emilie Brotin, Jérôme E. Lafont, Yannick Saintigny, Laurent Poulain, Christophe Denoyelle, Magali Demoor, Florence Legendre, Philippe Galéra

**Affiliations:** 1 Normandie Univ, UNICAEN, BIOTARGEN, 14000 Caen, France; clement.veys@unicaen.fr (C.V.); abderrahim.benmoussa@umontreal.ca (A.B.); contentinr@email.chop.edu (R.C.); aduchemin@uliege.be (A.D.); magali.demoor@unicaen.fr (M.D.); florence.legendre@unicaen.fr (F.L.); 2 Research Center of the UHC Sainte-Justine and Department of Nutrition, Université de Montréal, Montréal, QC H3T 1C54, Canada; 3 Normandie Univ, UNICAEN, ImpedanCELL Platform, Federative Structure 4206 ICORE, 14000 Caen, France; e.brotin@baclesse.unicancer.fr (E.B.); cdenoyelle@baclesse.unicancer.fr (C.D.); 4 Normandie Univ, UNICAEN, INSERM U1086 ANTICIPE, Biology and Innovative Therapeutics for Ovarian Cancer (BioTICLA), 14000 Caen, France; l.poulain@baclesse.unicancer.fr; 5 Unicancer, Comprehensive Cancer Center F. Baclesse, 14000 Caen, France; 6 CNRS UMR 5305, Laboratory of Tissue Biology and Therapeutic Engineering, Université Claude Bernard Lyon 1, Univ Lyon, 69367 Lyon, France; jerome.lafont@univ-lyon1.fr; 7 LARIA, iRCM, François Jacob Institute, DRF-CEA, 14000 Caen, France; yannick.saintigny@cea.fr; 8 Normandie Univ, ENSICAEN, UNICAEN, CEA, CNRS, UMR6252 CIMAP, 14000 Caen, France

**Keywords:** chondrosarcoma, miR-342-5p, miR-491-5p, Bcl-2/Bcl-xL, apoptosis, autophagy

## Abstract

Chondrosarcomas are malignant bone tumors. Their abundant cartilage-like extracellular matrix and their hypoxic microenvironment contribute to their resistance to chemotherapy and radiotherapy, and no effective therapy is currently available. MicroRNAs (miRNAs) may be an interesting alternative in the development of therapeutic options. Here, for the first time in chondrosarcoma cells, we carried out high-throughput functional screening using impedancemetry, and identified five miRNAs with potential antiproliferative or chemosensitive effects on SW1353 chondrosarcoma cells. The cytotoxic effects of miR-342-5p and miR-491-5p were confirmed on three chondrosarcoma cell lines, using functional validation under normoxia and hypoxia. Both miRNAs induced apoptosis and miR-342-5p also induced autophagy. Western blots and luciferase reporter assays identified for the first time Bcl-2 as a direct target of miR-342-5p, and also Bcl-xL as a direct target of both miR-342-5p and miR-491-5p in chondrosarcoma cells. MiR-491-5p also inhibited EGFR expression. Finally, only miR-342-5p induced cell death on a relevant 3D chondrosarcoma organoid model under hypoxia that mimics the in vivo microenvironment. Altogether, our results revealed the tumor suppressive activity of miR-342-5p, and to a lesser extent of miR-491-5p, on chondrosarcoma lines. Through this study, we also confirmed the potential of Bcl-2 family members as therapeutic targets in chondrosarcomas.

## 1. Introduction

Chondrosarcomas are the second-most common primary malignant bone tumors after osteosarcomas [1,2]. They mainly affect adults between 30 and 70 years old. Chondrosarcomas are an heterogeneous group of tumors characterized by the production of an extracellular matrix with cartilaginous characteristics [3]. They are classified into different histological grades, from low to high, related to their metastatic potential and associated survival rates. Chondrosarcomas are resistant to conventional radiotherapy and chemotherapy. Several mechanisms are involved in their resistance [4]. For instance, they are poorly vascularized, produce an abundant cartilaginous extracellular matrix and are composed of a limited number of proliferating cells which, as whole, hinder drug efficacy. The hypoxic microenvironment of chondrosarcomas is also a major contributor to their radio-resistance, because it prevents the formation of antineoplastic reactive oxygen species (ROS) after irradiation [4]. Therefore, lethality varies between 10% and 50%. Consequently, the only effective treatment for chondrosarcomas is extensive resection of the tumor [4]. However, surgery is not always feasible, especially for tumors located, for example, at the base of the skull. Therefore, there is an urgent need for new therapeutic strategies to treat chondrosarcomas and/or overcome their resistance to conventional therapies.

MicroRNAs (miRNAs) are small non-coding RNAs of about 20–25 nucleotides, which act as post-transcriptional regulators of mRNA, generally through base-pairing and a subsequent translation blockade or mRNA decay [5]. A single miRNA can regulate several hundred different mRNAs. Additionally, several miRNAs can share the same mRNA as a target, thereby allowing miRNAs to be key regulators of complex networks of targets [6,7]. MiRNAs play an essential role in many physiological processes, but also in numerous pathological conditions, particularly in cancer progression, including chondrosarcomas. Several studies have reported an aberrant expression of miRNAs in chondrosarcomas [8,9]. For example, miR-100, already identified as a tumor suppressor in many cancers, is downregulated in chondrosarcomas compared with normal articular chondrocytes [8]. Another study found that miR-100 is able to re-sensitize resistant chondrosarcoma cells to cisplatin through the direct targeting of mammalian target of rapamycin kinase (mTOR) [10]. Consequently, miRNAs can be used as diagnostic and prognostic biomarkers, but may also allow the discovery of novel therapeutic targets in chondrosarcomas [9,11,12]. Various pathways, regulated by miRNAs, that influence proliferation, progression, invasion, angiogenesis and chemosensitivity have been identified in chondrosarcomas. However, to the best of our knowledge, no study has explored the effect of miRNAs that directly target anti-apoptotic molecules such as B-cell lymphoma-2 (Bcl-2), Bcl-2 lymphoma-extra large (Bcl-xL) and Myeloid cell leukemia-1 (McL-1) in chondrosarcomas. Only two studies using siRNA designed to target anti-apoptotic molecules Bcl-2, Bcl-xL, X-linked inhibitor of Apoptosis Protein (XIAP) or survivin have demonstrated an increase in sensitivity to doxorubicin or irradiation [13,14]. The restoration of chemosensitivity to doxorubicin and cisplatin has also been obtained with the BH3 mimetic ABT-737 in various chondrosarcoma cell lines [15,16].

In the present study, using functional high-throughput miRNA screening and miRNA target prediction, we selected five miRNAs that can induce apoptosis in the SW1353 chondrosarcoma cell line by targeting *BCL2*, *BCL2L1* or *MCL1* mRNAs. Thereafter, we performed functional studies with the individual miRNAs: miR-149-5p, miR-342-5p, miR-491-5p, miR-541-5p and miR-625-5p. The potential cytotoxic and chemosensitizing effects of the miRNAs were studied under both normoxia and physioxia (hypoxia), the latter being more representative of the in situ physiopathological microenvironment of chondrosarcomas [17]. We validated the apoptotic effects of miR-491-5p and miR-342-5p in three chondrosarcoma cell lines in both oxic conditions and identified key signaling pathways involved in their activity. Using a luciferase assay, we demonstrated that miR-342-5p directly inhibits both anti-apoptotic *BCL2L1* and *BCL2* mRNAs post-transcriptionally, and miR-491-5p directly inhibits *BCL2L1* mRNA post-transcriptionally. Considering the importance of autophagy in cancer biology and the growing number of autophagy-related miRNAs [18], we also evaluated autophagy, and found that miR-342-5p can activate this process. Finally, we demonstrated for the first time the tumor suppressive effect of miR-342-5p in a 3D organoid grown under hypoxia, a culture model more representative of the physiopathology of chondrosarcomas than 2D cell cultures.

## 2. Results

### 2.1. High-Throughput Screening Identifies miRNAs with Potential Antiproliferative and Chemosensitive Effects on A Chondrosarcoma Cell Line

**Figure 1:**
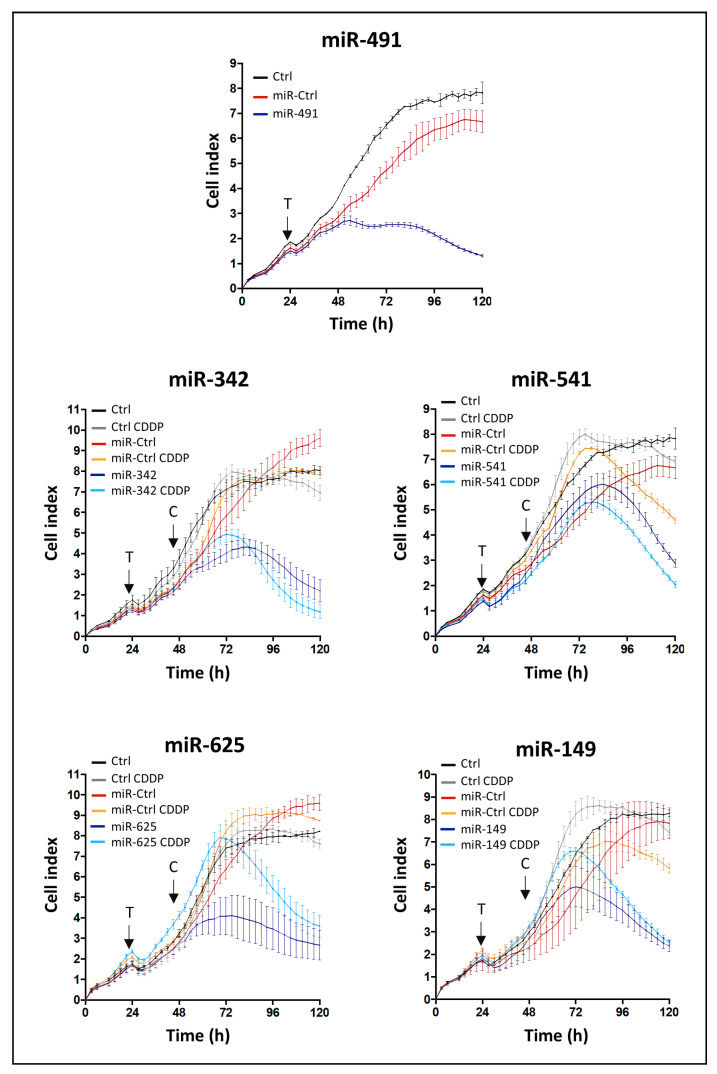
Identification of miRNAs with potential antiproliferative and chemosensitizing effects using high-throughput screening. SW1353 cells were seeded in triplicate in 96-well E-plates VIEW. They were grown for 24 h before transfection (arrow labeled with a T) with 20 nM of miR-Ctrl, miR-491-5p, miR-342-5p, miR-541-5p, miR-625-5p or miR-149-5p. The cells were also treated with 1 µg/mL of CDDP 24 h post-transfection or left untreated (arrow labeled with a C). Each condition was performed in triplicate. Real-time growth curves were monitored using the xCELLigence System. Impedance was recorded every 2 h for 120 h. Cell index profiles of one experiment per miRNA species, with mean ± SD, are shown. CDDP: cisplatin, Ctrl: control.

We carried out a functional high-throughput screening with a human library of 1200 miRNA mimics on the SW1353 chondrosarcoma cell line. The study was based on the continuous measurement of impedance to analyze the dynamic behavior of the cells (adhesion, proliferation and survival) after transfection with miRNA mimics combined with or without cisplatin treatment (CDDP). The effect of each miRNA was compared with that of the miR-Ctrl. We used three criteria to select the most potent cytotoxic miRNAs: the shape of the curve, the area under the curve (AUC) and the cell index (CI) at the end of the experiment (Figure 1). Moreover, morphological observation of cell confluence provided complementary information for the final selection of miRNAs (Figure S1). Finally, of all these miRNAs, five showed a significant effect on cell proliferation/attachment (Figure 1 and Figure S1).

MiR-491-5p, an “apoptomiR” previously identified as cytotoxic in ovarian cancer cells with the same xCELLigence cell analysis system [19], was initially used as a positive control. In SW1353 cells, compared with miR-Ctrl, miR-491-5p substantially altered the shape of the curve as early as 24 h after transfection. Consequently, at 96 h post-transfection, the cell index decreased by 4.4-fold relative to miR-Ctrl (*p* < 0.01, unpaired Student’s *t*-test), clearly demonstrating the potential cytotoxic effect of miR-491-5p on SW1353 cells.

MiR-342-5p modified the cell index from approximately 36 h post-transfection until the end of the experiment (5-fold decrease relative to miR-Ctrl, 120 h after plating the cells, *p* < 0.0001). In the presence of a sublethal dose of CDDP and miR-342-5p, the cell index decreased further (7-fold relative to miR-Ctrl + CDDP, 120 h after seeding, *p* < 0.001), suggesting that miR-342-5p may sensitize SW1353 cells to CDDP.

We observed similar results when combining miR-541-5p and CDDP with a final cell index lower than for miR-541-5p alone. However, the antiproliferative effect of miR-541-5p seemed less marked than that of miR-342-5p. It only decreased the cell index by 2.1-fold, 120 h after seeding (*p* < 0.01 relative to miR-Ctrl), and its effect was delayed by 28 h (started approximately 60 h post-transfection versus 32 h for miR-342).

MiR-625-5p and miR-149-5p also decreased the cell index from 36 h and 48 h post-transfection, respectively, indicating their antiproliferative activity. Compared with miR-Ctrl, the transfection of these miRNAs led to a 3.8- and 3.2-fold decrease in cell index, respectively, 120 h after seeding (*p* < 0.001 and *p* < 0.01, respectively). Cell indices were higher in the presence of CDDP for both miRNAs. The final cell index was the same for miR-149 with or without CDDP and higher for miR-625 with CDDP than without CDDP.

At the end of the experiment, morphological examination of the cells confirmed that all five miRNAs under study reduced cell proliferation compared with miR-Ctrl (Figure S1).

### 2.2. MiR-491-5p and miR-342-5p Have Antimetabolic and Cytotoxic Effects on the SW1353 Chondrosarcoma Cell Line

Next, we assessed the antiproliferative effects of these five selected miRNAs with a functional analysis performed in both normoxia and hypoxia on SW1353 cells.

**Figure 2:**
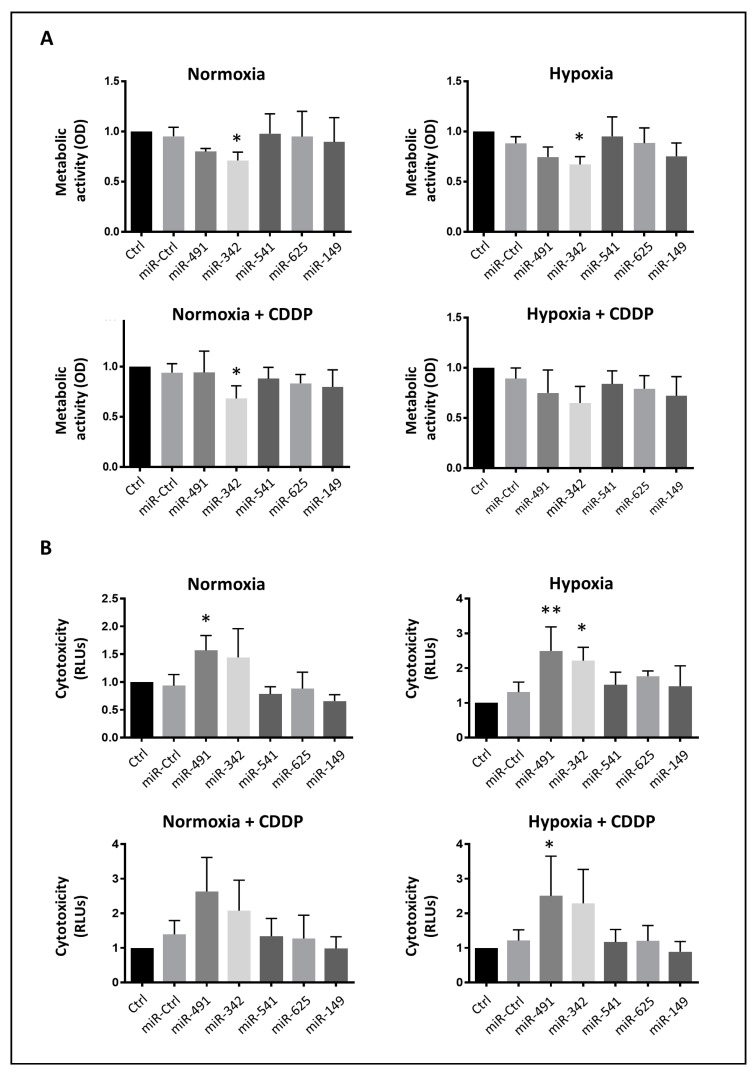
Analysis of the cytotoxic and antimetabolic effects of miRNAs on SW1353 cells. SW1353 cells were cultured under normoxia or hypoxia. They were transfected 24 h after seeding with 20 nM of miR-Ctrl, miR-491-5p, miR-342-5p, miR-541-5p, miR-625-5p or miR-149-5p. The treatment with CDDP (1 µg/mL) was performed 48 h post-transfection for 24 h. (**A**) The metabolic activity of cells was evaluated 72 h post-transfection and expressed as the mean optical density (OD) ± SD of five independent experiments. (**B**) The cytotoxicity of the miRNAs was evaluated 72 h post-transfection and expressed as the mean relative luciferase units (RLU) ± SD of four independent experiments. The significance of the results between miR-Ctrl and miRNA-treated cells was assessed using one-way ANOVA (*: *p* < 0.05, **: *p* < 0.01).

First, we carried out an XTT cell viability assay to measure the metabolic activity of the cells (Figure 2A). Only miR-342-5p significantly decreased cellular metabolism under normoxia and hypoxia (0.7-fold decrease relative to miR-Ctrl, *p* < 0.05). MiR-491-5p seemed to reduce metabolic activity (0.8-fold decrease), but this effect was not statistically significant.

Afterwards, we investigated the potential chemosensitizing effect of the five miRNAs of interest (Figure 2A). Upon sublethal exposure to CDDP, only the cells treated with miR-342-5p under normoxic conditions experienced a significant decrease in their metabolic activity (0.75-fold decrease relative to miR-Ctrl/CDDP-treated cells, *p* < 0.05). In all cases, the metabolic activity did not further decrease with the combination of miRNAs and CDDP, compared with miRNAs alone.

Then, we investigated the potential cytotoxic effects of these miRNAs (Figure 2B). Only miR-491-5p and miR-342-5p elicited significant cytotoxic effects on the SW1353 cells with a 1.7- and 1.9-fold increase in cytotoxicity for miR-491-5p under normoxia and hypoxia, respectively, and a 1.7-fold increase for miR-342-5p under hypoxia, compared with their respective miR-Ctrl. In the presence of CDDP, these miRNAs increased cytotoxicity by the same magnitude as without CDDP, suggesting no chemosensitizing effect. In this assay, we did not confirm the potential chemosensitizing effect of miR-342-5p and miR-541-5p expected from the high-throughput screening using the xCELLigence system.

### 2.3. MiR-491-5p and miR-342-5p Induce Cell Death in Three Chondrosarcoma Cell Lines, But Do Not Affect the Cell Cycle of Healthy Human Articular Chondrocytes

**Figure 3:**
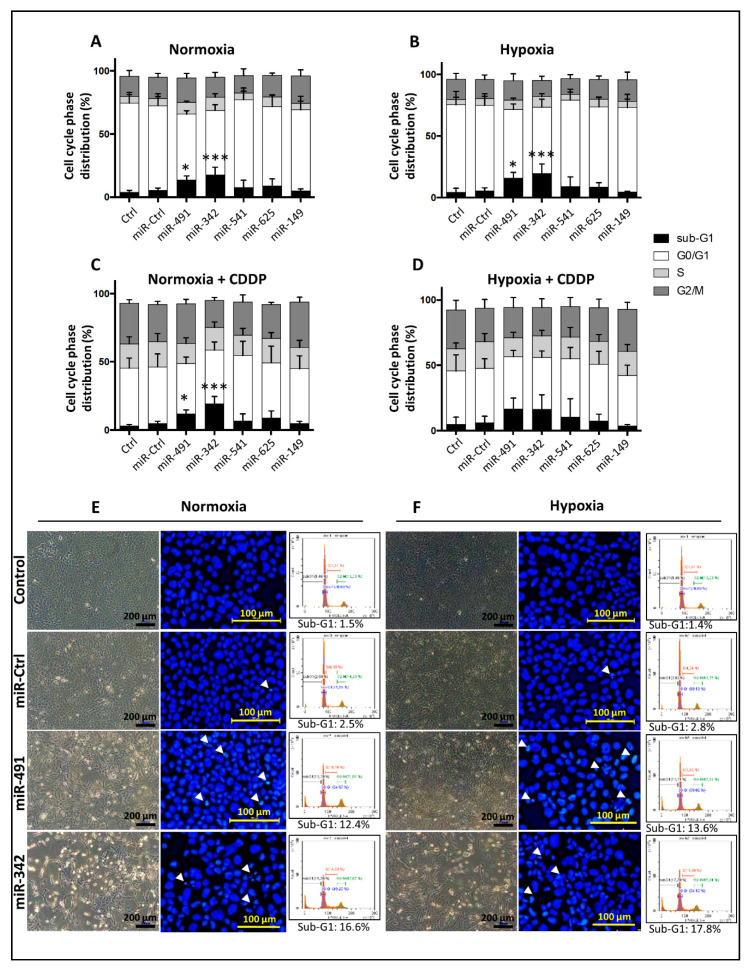
Functional validation of cytotoxic or chemosensitizing effects of individual miRNAs on SW1353 cells. SW1353 cells were cultured under normoxia (**A**,**C**,**E**) or hypoxia (**B**,**D**,**F**). They were transfected 24 h after seeding with 20 nM of miR-Ctrl, miR-491-5p, miR-342-5p, miR-541-5p, miR-625-5p or miR-149-5p. An additional treatment with cisplatin (CDDP) (1 µg/mL) was performed 48 h post-transfection (C and D). Analyses were carried out 72 h post-transfection. (**A**–**D**) Cell cycle phase distribution was analyzed using flow cytometry. The histograms represent the analysis of five independent experiments (mean ± SD) with the different phases of the cycle. Statistically significant differences in the percentage of sub-G1 events between miR-Ctrl and miRNA-treated cells were determined using one-way ANOVA (*: *p* < 0.05, ***: *p* < 0.001). (**E**,**F**) The left panels show the cell morphology obtained under photonic microscopy at the end of the experiment. The middle panels show nuclear morphology obtained after DAPI staining as described in the Materials and Methods section. White arrowheads show cells with condensed and/or fragmented chromatin and cellular debris. The right panels show DNA content histograms obtained using flow cytometry. Images shown are representative of five independent experiments.

We analyzed cell cycle progression using flow cytometry in the SW1353 chondrosarcoma cell line (Figure 3). None of the miRNAs under investigation induced a cell cycle blockade, but miR-491-5p and miR-342-5p induced a significant accumulation of cells in the sub-G1 phase associated with the induction of cell death.

Regardless of the oxic conditions, miR-491-5p and miR-342-5p increased the percentage of sub-G1 events, by 2.8-fold average increase under normoxia and by 3.4-fold average increase under hypoxia (*p* < 0.05 and *p* < 0.001 relative to miR-Ctrl-treated cells in each group; Figure 3A,B). There was also a decrease in cellular density, more cellular debris and condensed and/or fragmented chromatin—typical of apoptotic cells—after miR-491-5p and miR-342-5p transfection (Figure 3E,F).

The efficiency of the transfection of miR-541-5p, miR-625-5p and miR-149-5p was similar in extent to that observed for miR-491-5p and miR-342-5p (overexpression of at least 3000-fold, *p* < 0.001; Figure S2). However, their overexpression did not induce cell death (Figure 3A,B), the cells reached confluency and no cellular debris was detected (data not shown).

Treatment with a sublethal dose of CDDP increased the percentage of events in the S and G2/M phases at the expense of the G0/G1 phase (Figure 3C,D and Figure S3). MiR-491-5p and miR-342-5p increased the percentage of sub-G1 events when combined with CDDP, with significance reached only under normoxia (by 2.5-fold with *p* < 0.05 and by 4.1-fold with *p* < 0.01, respectively, relative to miR-Ctrl/CDDP-treated cells). The miRNA-induced increases in sub-G1 peaks were comparable with or without CDDP, suggesting no chemosensitizing effect for any of the miRNAs. From these results, we conclude that the antiproliferative effects of miR-491-5p and miR-342-5p were due to the induction of cell death in the SW1353 cell line, without any chemosensitizing effect.

**Figure 4:**
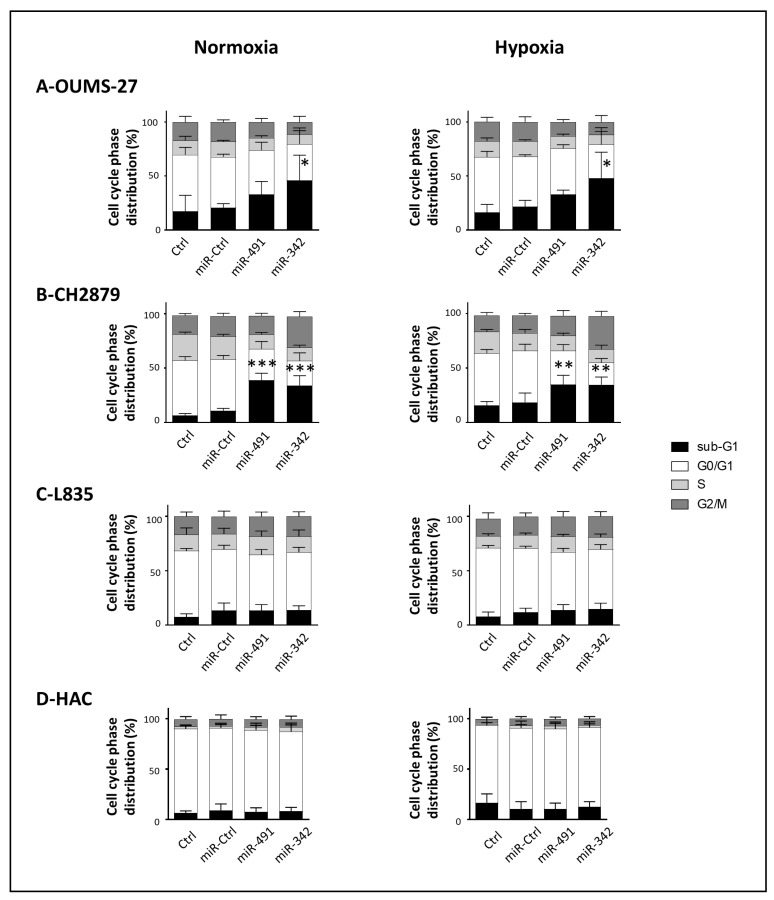
Analysis of the cytotoxic effects of miR-491-5p and miR-342-5p on chondrosarcoma cells and on HACs. OUMS-27 cells (**A**), CH2879 cells (**B**), L835 cells (**C**), and HACs (**D**) were cultured under normoxia or hypoxia. Cell lines were transfected 24 h after seeding with 20 nM of miR-Ctrl, miR-491-5p or miR-342-5p. Primary HACs were transfected with 20 nM miRNAs 5 days after seeding. Cell cycle phase distribution was analyzed using flow cytometry 72 h post-transfection. The histograms represent the analyses of the different phases of the cell cycle of at least four independent experiments (mean ± SD). Statistically significant differences in the percentage of sub-G1 events between miR-Ctrl and miRNA-treated cells were determined using one-way ANOVA (*: *p* < 0.05, **: *p* < 0.01, ***: *p* < 0.001).

We thus focused subsequent investigations on these two miRNAs and assessed their effects on three other chondrosarcoma cell lines (OUMS-27, CH2879 and L835) and on primary human articular chondrocytes (HACs) as healthy controls (Figure 4). In OUMS-27 cells, miR-491-5p increased the percentage of sub-G1 events under normoxia and hypoxia (by 1.6- and 1.5-fold, respectively, but this increase was not significant; Figure 4A). MiR-342-5p induced a 2.2-fold increase (*p* < 0.05) in the sub-G1 peak compared with miR-Ctrl-treated cells, under both oxic conditions. In CH2879 cells, miR-491-5p significantly increased the percentage of sub-G1 events (by 3.6-fold under normoxia (*p* < 0.001) and 1.9-fold under hypoxia (*p* < 0.01); Figure 4B). MiR-342-5p also raised the percentage of sub-G1 events (by 3.2-fold under normoxia (*p* < 0.001) and 1.9-fold under hypoxia (*p* < 0.01)). The increase in sub-G1 peaks with miR-491-5p and miR-342-5p, indicating induction of cell death in both cell lines, was supported by the extensive cellular debris and numerous apoptotic cells observed under fluorescence microscopy on DAPI-stained slides (Figure S4). In L835 cells, miR-491-5p and miR-342-5p did not induce any cell cycle arrest, nor did they significantly alter the sub-G1 peak, regardless of the oxic conditions (Figure 4C and Figure S5A). Therefore, these two miRNAs did not induce cell death in this chondrosarcoma cell line. We observed similar results for primary HACs transfected with miR-491-5p and miR-342-5p, in both oxic conditions, suggesting that these miRNAs have no cytotoxic effects on healthy cartilage cells (Figure 4D and Figure S5B).

In OUMS-27 and CH2879 cells, the treatment with a sublethal dose of CDDP significantly increased the percentage of events in the S phase under normoxia and hypoxia (from 1.5- to 2-fold; Figures S6A and S6B). In L835 cells, CDDP treatment increased the percentage of G2/M events: 1.9-fold in normoxia (*p* < 0.05) and 2.7-fold in hypoxia (*p* < 0.001) (Figure S6C). As in SW1353 cells, the miR-491-5p- and miR-342-5p-induced increases in sub-G1 peaks were similar with or without CDDP, suggesting no chemosensitizing effect of these miRNAs.

Overall, miR-491-5p and miR-342-5p seem to be apoptomiRs in SW1353, OUMS-27 and CH2879 chondrosarcoma cell lines, but do not appear to chemosensitize the cells to CDDP activity.

### 2.4. MiR-491-5p and miR-342-5p Activate the Apoptosis Pathway under Normoxia and Hypoxia in Chondrosarcoma Cells

**Figure 5:**
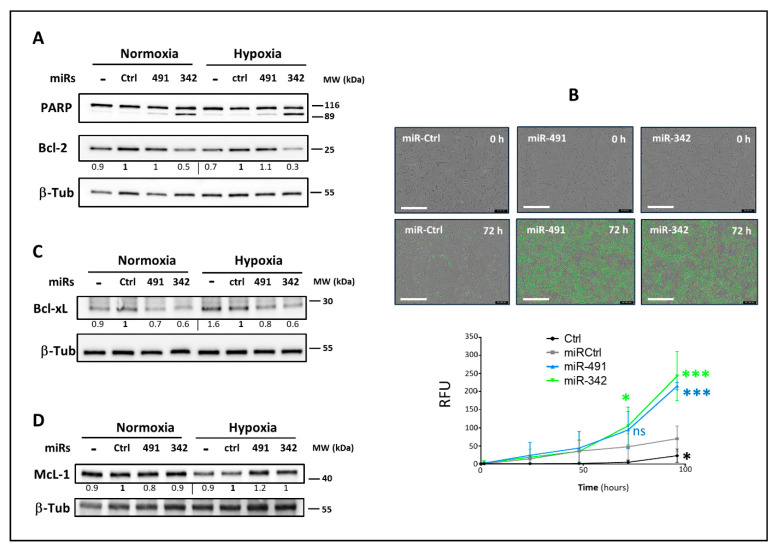
Analysis of the apoptotic effects of miR-491-5p and miR-342-5p on SW1353 cells. In parts (**A**,**C**,**D**), SW1353 cells were cultured under normoxia or hypoxia. They were transfected 24 h after seeding with 20 nM of miR-Ctrl, miR-491-5p or miR-342-5p. Analyses were carried out 72 h post-transfection. Protein extracts were analyzed with Western blots to evaluate PARP, Bcl-2, Bcl-xL and McL-1 levels versus a β-tubulin (β-Tub) loading control. Representative blots of three independent experiments are shown. Protein expressions are indicated at the bottom of the blots. They were normalized to β-tubulin and to the corresponding miR-Ctrl for each oxic condition. (**B**) SW1353 cells were cultured under normoxia and transfected 24 h after seeding as before. Caspase-3/7 activity was then assessed in real time for 96 h, as described in the Materials and Methods section. The images shown are representative of the experimental results obtained. Scale bar: 400 µm. The graph provides the analysis of fluorescence of four independent experiments (mean RFU ± SD). The significance of the results between miR-Ctrl and miRNA-treated cells was assessed using two-way ANOVA (*: *p* < 0.05, ***: *p* < 0.001).

We then focused our investigations on the induction of apoptosis. Regardless of the oxygen level, miR-491-5p and miR-342-5p induced the cleavage of Poly(ADP-Ribose) Polymerase (PARP), with more marked effects of miR-342-5p on SW1353 cells (Figure 5A), which is in accordance with our previous results on cell death (Figure 3). PARP was also cleaved in OUMS-27 and CH2879 cell lines, in both oxic conditions (Figure S7A). In addition, we evaluated caspase-3/7 activity in SW1353 cells by measuring green fluorescence in the nuclei, using the Incucyte^®^ Caspase-3/7 Green apoptosis assay system.

Both miRNAs increased caspase-3/7 activity in SW1353 cells, with higher significance observed 96 h post-transfection (*p* < 0.001, Figure 5B). Because PARP cleavage by activated caspases is also a hallmark of apoptosis, these two results imply that miR-491-5p and miR-342-5p induce apoptosis in chondrosarcoma cells.

Members of the anti-apoptotic Bcl-2 family are among the predicted targets for both miRNAs (Bcl-xL (validated) and McL-1 for miR-491-5p; Bcl-2, Bcl-xL (validated) and McL-1 for miR-342-5p, Tables S1 and S2). We therefore evaluated their expression to assess their involvement in the induction of apoptosis by these miRNAs in chondrosarcoma cell lines.

Bcl-2 protein expression greatly decreased upon miR-342-5p overexpression in SW1353 cells (by 2-fold under normoxia and 3.3-fold under hypoxia compared with their respective miR-Ctrl, Figure 5A). We obtained comparable results for OUMS-27 and CH2879 cells (Figure S7B). In contrast, miR-491-5p did not modulate the expression of Bcl-2 in SW1353 (Figure 5A) or OUMS-27 cells (Figure S7B), independently of the oxic conditions, whereas it decreased Bcl-2 protein levels by approximately 2-fold in CH2879 cells under hypoxia (Figure S7B). MiR-491-5p and miR-342-5p decreased Bcl-xL protein expression in SW1353 cells under both oxic conditions (from 1.3- to 1.7-fold, Figure 5C). Both miRNAs also reduced Bcl-xL protein levels in OUMS-27 and CH2879 cells (Figure S7C). Although McL-1 may be a potential target of these two miRNAs, they had no effect on its expression in either SW1353 (Figure 5D) or OUMS-27 cells (Figure S7D). On the contrary, they tended to increase McL-1 protein levels in CH2879 cells (in particular with miR-491-5p, Figure S7D).

In summary, miR-342-5p seems able to limit the expression of Bcl-2 and Bcl-xL, and miR-491-5p seems to be a negative regulator of Bcl-xL, effects that underlie their pro-apoptotic activity in chondrosarcoma cells.

### 2.5. MiR-342-5p Targets BCL2L1 and BCL2 mRNAs, Whereas miR-491-5p Targets Only BCL2L1 mRNA in the SW1353 Chondrosarcoma Cell Line

To verify that miR-491-5p and miR-342-5p effects on Bcl-2 (*BCL2* gene) or BCL-xL (*BCL2L1* gene) were through direct binding to their respective mRNAs, we co-transfected luciferase-reporter vectors containing the 3′Untranslated Region (UTR) sequence of these mRNAs along with either miR-491-5p or miR-342-5p mimics or their corresponding hairpin inhibitors in SW1353 cells.

**Figure 6:**
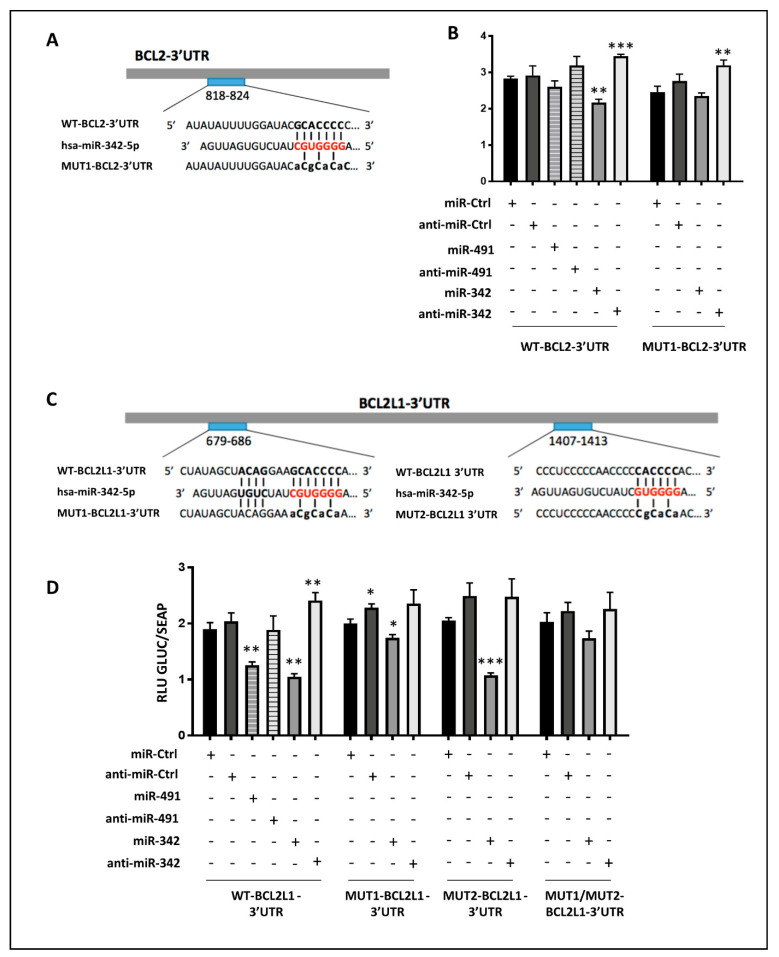
Inhibition of both *BCL2* and *BCL2L1* mRNAs by miR-342-5p and of *BCL2L1* mRNA by miR-491-5p. (**A**) The 3’UTR sequence of *BCL2* was aligned with the conserved sequence complementary to hsa-miR-342-5p. Wild (WT) and mutant (MUT1) luciferase vectors of BCL2-3’UTR are shown. (**B**) WT and MUT1 reporter vectors of BCL2-3’UTR (0.25 ng/µL) were co-transfected with the indicated miR or anti-miR (20 nM) in SW1353 cells. (**C**) The 3’UTR sequences of *BCL2L1* were aligned with the conserved sequence targeted by hsa-miR-342-5p. WT and mutants (MUT1, MUT2) luciferase vectors including BCL2L1-3’UTR are shown. The MUT1/MUT2-BCL2L1-3’UTR vector contains both mutations 1 and 2. (**D**) WT and mutant vectors of BCL2L1-3’UTR (1 ng/µL) were co-transfected with the indicated miR or anti-miR (20 nM) in SW1353 cells. (**B**,**D**) SEAP and GLUC luciferase activities were measured 24 h post-transfection. GLUC activity was normalized to SEAP activity and expressed as RLU. The data show a representative experiment (mean ± SD) of four independent experiments performed in triplicate. The significance of the results between miR-Ctrl and miRNA- or anti-miRNA-treated cells was evaluated by two-tailed Student’s *t*-test (*: *p* < 0.05, **: *p* < 0.01, ***: *p* < 0.001).

In silico analysis identified a potential binding site for miR-342-5p between nucleotides 818 and 824 in the 3′UTR of BCL2 (BCL2-3’UTR; Figure 6A). Only the transfection of miR-342-5p significantly decreased the luciferase activity (RLU) of the reporter vector containing the wild-type (WT) BCL2-3′UTR (−23% relative to miR-Ctrl, *p* < 0.01, Figure 6B). In contrast, the co-transfection with anti-miR-342-5p significantly increased RLU (by 22%), which is consistent with an inhibitory effect of miR-342-5p. Mutation in the binding site for miR-342-5p in the 3′UTR of BCL2 prevented the inhibitory effect of this miRNA. As previously, co-transfection with anti-miR-342-5p increased RLU (+30%, *p* < 0.01).

In silico analysis did not identify any potential binding site for miR-491-5p in the 3′UTR of *BCL2* mRNA. As expected, miR-491-5p did not modulate the luciferase activity of the WT-BCL2-3′UTR reporter construct (Figure 6B). Therefore, in SW1353 cells, only miR-342-5p can downregulate Bcl-2 protein expression through direct binding to the 818–824 region in the 3′UTR of *BCL2* mRNA.

We identified two putative binding sites for miR-342-5p in the 3′UTR of BCL2L1 at positions 679–686 and 1407–1413. We thus made constructs in which these sites were mutated separately (MUT1 and MUT2 respectively, Figure 6C). Transfection of miR-342-5p led to a significant decrease in luciferase activity only when co-transfected with WT-BCL2L1-3′UTR or MUT2-BCL2L1-3′UTR (−45% and −47%, *p* < 0.01 and *p* < 0.001, respectively; Figure 6D). In comparison, the overexpression of this miRNA decreased reporter activity by only 13% (*p* < 0.05) for the MUT1-BCL2L1-3′UTR construct and by 14% (NS) for the double mutant MUT1/MUT2-BCL2L1-3′UTR construct. In parallel, co-transfection of anti-miR-342-5p with all the constructs led to a slight increase in luciferase activities. Therefore, in SW1353 cells, miR-342-5p downregulates Bcl-XL protein expression through preferential and direct binding to the 679–686 region in the 3′UTR of *BCL2L1* mRNA.

Functional binding sites for miR-491-5p have previously been identified in the 3′UTR of BCL2L1 mRNA [19,20,21]. Accordingly, co-transfection of miR-491-5p and WT-BCL2L1-3′UTR, bearing three binding sites for miR-491-5p, led to a significant decrease in the luciferase activity of the reporter vector (−34% relative to miR-Ctrl, *p* < 0.01, Figure 6D). This repression was specifically abolished with anti-miR-491-5p. Therefore, as previously reported in other cancer cells, BCL2L1 mRNA is also a direct target of miR-491-5p in the SW1353 chondrosarcoma cell line.

### 2.6. MiR-342-5p Increases Autophagy in the SW1353 Chondrosarcoma Cell Line

**Figure 7:**
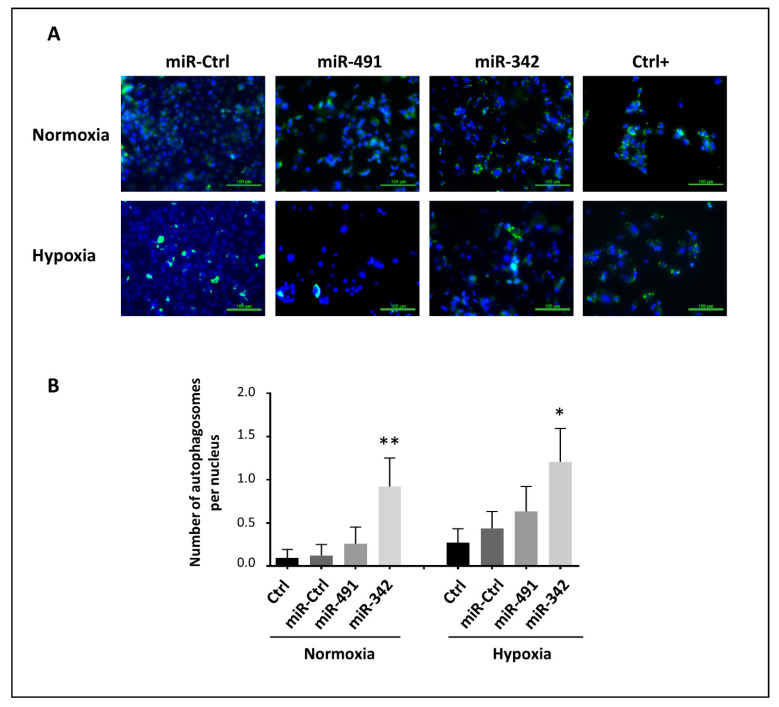
Effects of miR-491-5p and miR-342-5p on the autophagic activity of SW1353 cells. SW1353 cells were cultured and transfected as described in Figure 5, with an additional positive control (5% FCS in PBS for the last 24 h). Analyses were carried out 72 h post-transfection. The autophagosomes (green) and the nuclei (blue) were labeled as described in the Materials and Methods section. (**A**) The images shown are representative of the experimental results obtained. Scale bar: 100 µm. (**B**) The graph gives the number of autophagosomes normalized to the number of nuclei (mean ± SD of three independent experiments). The significance of the results between miR-Ctrl and miRNA-treated cells was assessed using one-way ANOVA (*: *p* < 0.05, **: *p* < 0.01).

To further investigate the mechanisms of action of miR-342-5p and miR-491-5p, we then investigated their ability to regulate autophagy, which can also contribute to cell death. We quantified the number of autophagosomes per nucleus using the specific autophagosome green marker of the Cell Meter^TM^ autophagy fluorescence imaging kit and DAPI staining of the nuclei (Figure 7).

Whatever the oxic conditions, miR-491-5p did not significantly modulate the number of marked autophagosomes (Figure 7). MiR-342-5p significantly increased the number of autophagosomes under normoxia (by 7.4-fold relative to miR-Ctrl-treated cells, *p* < 0.01), and to a lesser extent under hypoxia (by 2.8-fold, *p* < 0.05). Therefore, miR-342 increases the autophagic activity of SW1353 cells, which may participate in its tumor-suppressive activity.

### 2.7. MiR-491-5p and miR-342-5p Modulate the Expression of Numerous Proteins Related to Proliferation or Apoptosis and Affect Mitogenic Signaling Pathways in Chondrosarcoma Cells

To gain further insight into the mechanisms underlying the activity of these two miRNAs, we looked at the expression of some of their predicted, or validated, targets (Tables S1 and S2), with a special focus on those involved in cell survival/death and/or in signaling pathways commonly altered in cancer.

**Figure 8:**
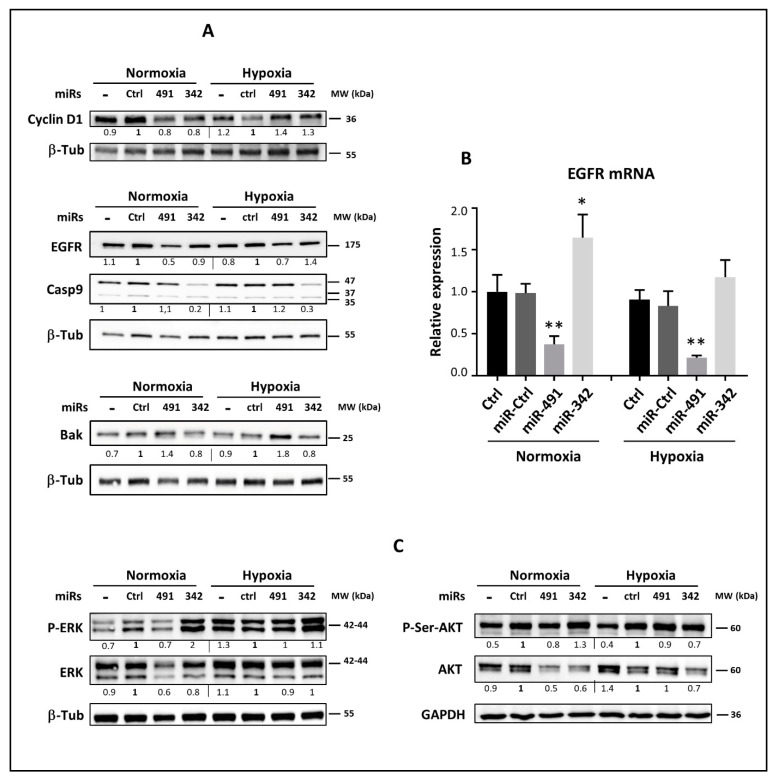
Analysis of some survival/death proteins and mitogenic signaling pathways affected by miR-491-5p and miR-342-5p. SW1353 cells were cultured and transfected as described in Figure 5. Analyses were carried out 72 h post-transfection. (**A**,**C**) Protein extracts were analyzed with Western blots to evaluate the levels of Cyclin D1, EGFR, Caspase-9, Bak, P-Thr202/Tyr204-ERK, ERK, P-Ser473-AKT and AKT versus β-tubulin (β-Tub) or GAPDH. Representative blots of three independent experiments are shown. Protein expressions are indicated at the bottom of the blots. They were normalized to β-tubulin or GAPDH, and to the corresponding miR-Ctrl for each oxic condition. (**B**) Total RNA was extracted as described in the Materials and Methods section. Relative expression of *EGFR* was determined by RT-quantitative PCR and normalized to both *PPIA1* and *GAPDH* levels and to Ctrl. Data are expressed as mean of triplicate samples ± SD. The results are representative of three independent experiments. The significance of the results was evaluated by two-tailed Student’s *t*-test compared with the corresponding miR-Ctrl (*: *p* < 0.05, **: *p* < 0.01).

Although in silico analysis showed that *CCND1* mRNA was a validated target of miR-342-5p (Table S2), the transfection of this miRNA did not clearly affect CCND1 protein expression in chondrosarcoma cell lines. In SW1353 cells, miR-491-5p and miR-342-5p led to a slight decrease in cyclin D1 protein expression under normoxia, whereas they tended to increase its levels under hypoxia (Figure 8A). We did not detect any reproducible alteration in cyclin D1 expression in the *CCND1* mRNA steady-state levels (data not shown). In CH2879 and OUMS-27 cells, there was no significant change in cyclin D1 expression independently of the oxic condition (Figure S8A).

We then measured the expression of *EGFR*, a direct target of miR-491-5p in glioblastoma and ovarian cancer cells [19,22] (Table S1), and a predicted target of miR-342-5p (Table S2). In SW1353 cells, miR-491-5p repressed the expression of *EFGR* at the mRNA level by 2.5-fold (*p* < 0.01) under both oxic conditions, whereas miR-342-5p increased its expression by 1.6-fold (*p* < 0.05) only under normoxia (Figure 8B). At the protein level, miR-491-5p decreased EGFR expression from 1.4- to 2-fold depending on the oxic condition (Figure 8A). This effect on EGFR protein was more marked in OUMS-27 and CH2879 chondrosarcoma cell lines than in SW1353 cells (Figure S8B). MiR-342-5p did not have any significant effect on EGFR protein expression in any of the three chondrosarcoma cell lines.

Caspase-9 was also identified as a direct target of miR-342-5p ([23], Table S2). MiR-342-5p did not significantly modulate caspase-9 mRNA level in SW1353 cells (data not shown). However, in the three chondrosarcoma cell lines, it caused a strong decrease (at least 3-fold) in pro-caspase-9 protein expression (47 kDa), without induction of the large cleaved caspase-9 fragments (37 and 35 kDa) (Figure 8A and Figure S8B). MiR-491-5p had no effect on pro-caspase-9 protein expression and it did not increase the cleavage of caspase-9.

The pro-apoptotic proteins Bcl-2-associated X (Bax) and Bcl-2 homologous antagonist killer (Bak) are essential for mitochondrial outer membrane permeabilization and induction of cell death. None of the considered miRNAs affected Bax protein expression (data no shown). Bak protein was upregulated when exposing the three chondrosarcoma cell lines to miR-491-5p, especially under hypoxia, whereas miR-342-5p tended to decrease Bak expression (from 1.3-fold to 3-fold increase with miR-491-5p and approximately 1.2-fold decrease with miR-342-5p, Figure 8A and Figure S8C).

Finally, we analyzed the effects of these miRNAs on two essential mitogenic signaling pathways involved in cell survival (Mitogen Activated Protein Kinase/Extracellular signal-Regulated Kinase (MAPK/ERK) and Protein Kinase B/AKT (PKB/AKT). In the ERK pathway, the effects of these miRNAs were quite variable from one cell line to another, and depended on the oxic conditions. In SW1353 cells, miR-491-5p decreased activated and total ERK proteins under normoxia (by approximately 1.5-fold, Figure 8C). Mir-491-5p had no marked effect on the ERK pathway in OUMS-27 cells (Figure S8D). It decreased phosphorylated or total ERK proteins levels under normoxia and hypoxia in CH2879 cells (maximum decrease of 1.6-fold for phosphorylated ERK under normoxia; Figure S8D). These results suggest that miR-491-5p can decrease the expression of ERK proteins and their activated/phosphorylated forms in chondrosarcoma cells. In SW1353 cells, miR-342-5p did not alter total ERK protein expression (Figure 8C). However, it increased the levels of phosphorylated ERKs under normoxia. In OUMS-27 and CH2879 cells, miR-342-5p did not seem to significantly influence total or phosphorylated ERK proteins levels (Figure S8D). Therefore, miR-342-5p does not significantly influence ERK signaling pathway in chondrosarcoma cells.

The effects of miRNAs on the AKT signaling pathway were also heterogeneous among the chondrosarcoma cell lines. In SW1353 cells, miR-491-5p decreased total AKT protein only under normoxia (by 2-fold, relative to miR-Ctrl-treated cells; Figure 8C), without clearly inhibiting AKT activation. MiR-342-5p downregulated the expression of total AKT protein under both normoxia and hypoxia (by approximately 1.5-fold), with subsequent inhibition of AKT phosphorylation only under hypoxia (by 1.4-fold). In OUMS-27 cells, there was no significant variation in phosphorylated or total AKT proteins levels between either miRNA or under either oxic condition (Figure S8E). In CH2879 cells, whereas both miR-342-5p and miR-491-5p decreased total AKT protein expression (from 1.5- to 5-fold), they also increased the level of phosphorylated AKT under normoxia and hypoxia (Figure S8E). Consequently, these results suggest that the effects of miR-491-5p and miR-342-5p on AKT signaling depend on the chondrosarcoma cell line. They can decrease AKT expression, but at the same time participate in its activation.

### 2.8. Only miR-342-5p Induces Cell Death in a 3D Organoid Chondrosarcoma Model

**Figure 9:**
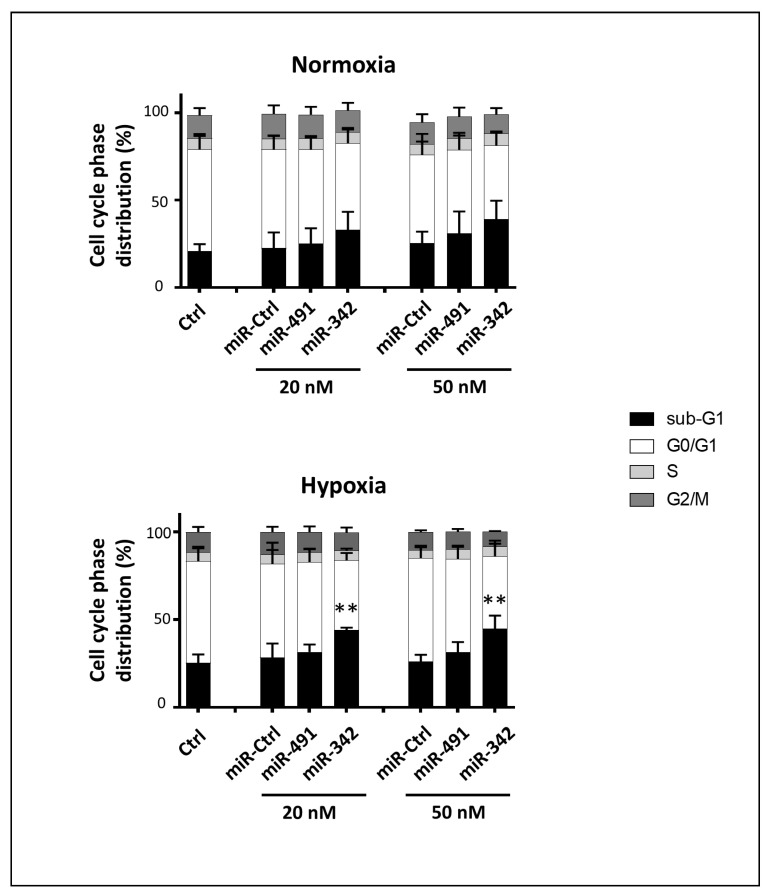
Effects of miR-491-5p and miR-342-5p on SW1353 cells cultured in 3D. SW1353 cells were cultured in collagen sponge scaffolds under normoxia or hypoxia. They were transfected 24 h after seeding with 20 nM or 50 nM of miR-Ctrl, miR-491-5p and miR-342-5p. Analyses were carried out 72 h post-transfection. Cell cycle phase distribution was analyzed using flow cytometry. The histograms represent the analysis of the different phases of the cell cycle of four independent experiments (mean ± SD). Statistically significant differences in the percentage of sub-G1 events between miR-Ctrl and miRNA-treated cells were determined using one-way ANOVA (**: *p* < 0.01).

Finally, we tested the anti-tumor activity of miR-342-5p and miR-491-5p on SW1353 cells cultured in 3D, a model that mimics the in vivo physiopathological microenvironment more accurately. SW1353 cells were transfected with either 20 or 50 nM of miRNA mimics. Cells were cultured under normoxia or hypoxia with the same kinetics as described above (Figure 9).

Compared with miR-Ctrl, miR-491-5p did not significantly increased the percentage of sub-G1 events under normoxia and hypoxia (by 1.1- and 1.2-fold, respectively, regardless of its concentration. In contrast, miR-342-5p increased the number of sub-G1 peaks, with the same efficiency at 20 as at 50 nM (approximately 1.5-fold relative to the respective miR-Ctrl, only significant under hypoxia at *p* < 0.01). More cellular debris and apoptotic cells were observed under fluorescence microscopy on DAPI-stained slides with miR-342-5p than with miR-491-5p (Figure S9). In conclusion, miR-342-5p, but not miR-491-5p, significantly induces cell death in our organoid chondrosarcoma culture model, especially in hypoxia.

## 3. Discussion

The resistance of chondrosarcomas to conventional radiotherapy and chemotherapy calls for the development of new treatments. The use of therapeutic miRNAs offers the advantage of a multi-target approach and can help to discover new therapeutic targets for clinical applications [9,11]. In previous studies, the functions of about 20 miRNAs have been reported in chondrosarcomas [9,24]. Based on high-throughput screening of a human library of 1200 miRNAs, we found numerous miRNAs able to decrease cell proliferation in the SW1353 chondrosarcoma cell line. Among these, we identified five miRNAs with putative pro-apoptotic activity because they potentially target anti-apoptotic proteins such as Bcl-xL and Bcl-2, which are thought to play a critical role in the chemoresistance of chondrosarcoma chondrocytes [15,16,25]. Moreover, two miRNAs (miR-342-5p and miR-541-5p) also seemed to exert chemosensitizing effects when combined with CDDP. Real-time cell analyses and endpoint morphological analyses are frequently performed in glass culture plates under normoxia, but chondrosarcoma cells live in a hypoxic environment. Therefore, we carried out functional analyses in standard plastic culture plates to validate our approach and to unambiguously identify miRNAs with realistic cytotoxic and chemosensitizing effects. Considering the role of hypoxia in the resistance of tumor cells to conventional treatments [4,26], we performed functional analyses under both normoxia (21% O_2_) and hypoxia (3% O_2_). We validated the tumor-suppressive effects of miR-491-5p and miR-342-5p on three chondrosarcoma cell lines cultured in monolayer. Their effects have already been described in other cancers, but to date, our work is the first to identify miRNAs acting as tumor suppressors in a hypoxic microenvironment. Moreover, because the traditional 2D cell culture does not reflect the in situ physiopathological microenvironment, we used for the first time a 3D organoid culture model to further confirm the effects of miRNAs on SW1353 cells.

We used four cell lines derived from central conventional chondrosarcomas of grade II (SW1353) and grade III (OUMS-27, CH2879 and L835). They are all resistant to CDDP: CH2879 and OUMS-27 are the least resistant (inhibitory concentration to reach 50% reduction in cell viability (IC_50_), 40 µM and 50 µM, respectively), L835 has an IC_50_ value of at least 200 µM and SW1353 is the most resistant with an IC_50_ of at least 400 µM [15]. Mutation analysis performed on TP53 and on isocitrate dehydrogenase (IDH) 1 or 2 has revealed distinct characteristics: SW1353 harbors both IDH2 and TP53 mutations, OUMS-27 harbors a TP53 mutation, CH2879 is wild type for IDH and TP53 mutations and L835 harbors an IDH1 mutation [27,28]. Only the L835 cell line did not respond to miR-491-5p and miR-342-5p, and harbors an IDH1 mutation. Gain-of-function mutations of IDH1/2 are found in 38–70% of primary central chondrosarcomas and only lead to DNA hypermethylation. Moreover, direct inhibition of the IDH1 mutant enzyme does not change the tumorigenic properties of chondrosarcoma cell lines in vitro [29]. The four cell lines express Bcl-2 and Bcl-xL proteins at different levels [15,25]. These latter two studies showed that L835 cells express higher levels of Bcl-xL protein than Bcl-2 protein. Moreover, inhibition of Bcl-xL and Bcl-2 with the ABT-737 drug reduces cell viability and induces apoptosis of L835 cells after 72 h of incubation [15]. Inhibition of Bcl-xL by both miR-491-5p and miR-342-5p should therefore have resulted in cell death by apoptosis. Complementary experiments would be necessary to verify inhibition of Bcl-xL by both miR-491-5p and miR-342-5p on L835 cells.

Unlike miR-541-3p [30,31], miR-541-5p has never been studied in cancers. MiR-625-5p and miR-149-5p have been previously reported to have tumor suppressive roles in various cancers, alone or combined with various chemotherapeutic agents [32,33,34,35,36]. Even if miR-149-5p seems to play a dual role in cancer [37], both miR-625-5p and miR-149-5p can induce apoptosis. Our screening showed that these three miRNAs likely have antiproliferative effects, but we could not confirm any significant antimetabolic, cytotoxic or killer effects on the SW1353 chondrosarcoma cell line. Furthermore, we did not validate the chemosensitizing effect of miR-541-5p 72 h post-transfection. Because cells growing on glass are more sensitive than cells growing on plastic, it is possible that killer effects require a longer incubation period in standard cell culture conditions, possibly more than 96 h post-transfection.

MiR-491-5p has already been described as a tumor suppressor in various cancers and several of its direct targets have been identified and include Bcl-xL in colorectal cancer [21], Bcl-xL and EGFR in ovarian cancer [19], Bcl-xL and TP53 in pancreatic cancer [20], Bcl-xL, EGFR and CDK6 in glioblastoma [22], Pyruvate Kinase M2 (PKM2) [38] and Forkhead box Protein P4 (FOXP4) in osteosarcoma [39], the most common type of bone cancers. In our study, miR-491-5p was first used as a positive control of cytotoxicity for the high-throughput screening. Given its high therapeutic potential, we next wanted to confirm its tumor suppressive effect on chondrosarcoma cell line, which has never been studied. We found it was able to induce cell death in SW1353, OUMS-27 and CH2879 chondrosarcoma cell lines cultured in monolayer under normoxia and hypoxia.

EGFR is constitutively activated in high-grade chondrosarcoma tumors [40]. Regardless of the oxygen level, miR-491-5p inhibited EGFR at the protein and mRNA levels. This inhibition suggests a possible downstream inhibition of the AKT and MAPK signaling pathways, leading to a decrease in chondrosarcoma cell proliferation and migration, as shown in other studies investigating the inhibition or silencing of EGFR in chondrosarcomas [40]. Previous studies have shown that the downstream inhibition of MAPK and AKT activity by miR-491-5p depends on the type of cancer or the cell line. In the SW1990 pancreatic cell line, miR-491-5p inhibits PI3K/AKT, but not RAS/MAPK [20]. This miRNA also inhibits AKT and MAPK signaling pathways in IGROV1-R10 ovarian cancer cells, whereas SKOV3, another ovarian cell line, maintains its AKT and MAPK activity [19]. In the present study, the effect of miR-491-5p on AKT and MAPK signaling pathways was heterogeneous among the chondrosarcoma cell lines and also depended on the oxic conditions.

MiR-491-5p did not influence the expression of the Bcl-2 anti-apoptotic protein in any of the three investigated chondrosarcoma cell lines sensitive to miR-491-5p. Similarly, although the anti-apoptotic McL-1 may be a potential target of miR-491-5p, this miRNA did not influence McL-1 protein expression in SW1353 and OUMS-27 cell lines, and rather tended to increase McL-1 expression in CH2879 cells, as observed in the IGROV1-R10 ovarian cell line [19]. Like others, we found that miR-491-5p directly targets *BCL2L1* mRNA [19,20,22]. Consequently, miR-491-5p decreased the expression of the anti-apoptotic protein Bcl-xL in the three chondrosarcoma cell lines responding to miR-491-5p under both oxic conditions. It also increased the expression of the Bak pro-apoptotic protein. Both these events may therefore contribute to its apoptotic activity in chondrosarcoma cells.

The miR-342-5p mechanism of action has been less investigated than that of miR-342-3p and, to our knowledge, never in chondrosarcomas. It has been identified as a tumor suppressor in neuroblastomas [30]. It inhibits colon cancer tumorigenesis through direct targeting of the N-a-acetyl transferase 10 protein (NAA10) and also promotes apoptosis [41]. In osteosarcomas, miR-342-5p inhibits cell growth, migration and invasion, and restores sensitivity to doxorubicin through direct targeting of Wnt member 7B (WnT7b) [42]. In the present study, high-throughput screening revealed that miR-342-5p has some antiproliferative effects on SW1353 cells. However, miR-342-5p did not significantly affect AKT and ERK mitogenic signaling pathways as previously reported for breast cancer cells [43], or *AKT* mRNA in inflammatory macrophages [44]. We observed AKT inhibition at the protein level in two out of the three cell lines (SW1353, CH2879), but at the same time the phosphorylation of AKT increased in CH2879 cells. There is evidence in the literature that miR-342-5p increases the level of phosphorylated forms of AKT via targeting a phosphatase on cardiomyocytes [23]. Others have shown that miR-342-5p attenuates the protein level of total AKT, whereas its phosphorylated protein level is stable in neural stem cells [45]. In view of our results, we cannot assume that the antiproliferative effects of miR-342-5p are linked to AKT and ERK pathways, but are likely linked to its pro-apoptotic effects. Indeed, miR-342-5p induced cell death and apoptosis in SW1353, OUMS-27 and CH2879 cells, independently of the oxygen level. In the three chondrosarcoma cell lines, miR-342-5p inhibited the expression of the anti-apoptotic proteins Bcl-2 and Bcl-xL, but not that of McL-1, with a more sustained effect under hypoxia. We also reported for the first time that miR-342-5p downregulates Bcl-2 protein expression by directly binding to sequence 818–824 of BCL2-3′UTR. Like Soriano et al. [30], we found that miR-342-5p directly targets the *BCL2L1* gene and in addition, we identified its binding site at position 679–686 of BCL2L1-3′UTR. Soriano et al. also identified the *CCND1* gene as a direct target of miR-342-5p in neuroblastomas, but in our experiments on chondrosarcoma cells lines, cyclin D1 protein expression was not affected by miR-342-5p.

Surprisingly, in the three chondrosarcoma cell lines, miR-342-5p also had anti-apoptotic effects, because it tended to decrease (by 1.2-fold) the protein expression of the Bak pro-apoptotic protein, and that of the pro-caspase-9 (at least 3-fold). Our data are in line with those of Hou et al., who identified exosomal miR-342-5p as a key cardioprotective molecule inhibiting apoptotic signaling via direct targeting of caspase-9 and Jun Kinase 2 (JNK2) in cardiomyocytes [23]. Caspase-9 activation is stimulated by dimerization instead of cleavage within the apoptosome. Dimerization facilitates autocatalytic cleavage, which results in the stabilization of the dimer [46]. Caspase-9 showed complete activity in its uncleaved form [47]. This caspase initiates apoptosis by cleaving and thereby activating executioner caspases-3, -6 and -7. In our study, cleaved caspase-9 was not clearly observed in all chondrosarcoma cell lines, even with miR-491-5p, whose activation of the intrinsic apoptosis pathway has already been demonstrated [19,20]. Despite anti-apoptotic potential, miR-342-5p clearly induced apoptosis as demonstrated by the induction of cleaved PARP and caspase-3/7 activity.

Autophagy is a lysosomal degradation pathway that protects cells from deleterious cytoplasmic components. Autophagy can also be associated with cell death [48,49]. Here, we found that miR-342-5p induced autophagy in SW1353 cells. The effect was less significant in hypoxia, probably because autophagy was already activated in a hypoxic environment. We also report the inhibition of Bcl-2 and Bcl-xL by miR-342-5p, which may contribute to the activation of autophagy. Indeed, Bcl-2 family proteins can inhibit autophagy by targeting and inhibiting Atg6/Beclin 1, which has an essential role in the formation of autophagosomes [50,51,52]. Inhibition of Bcl-xL by miR-491-5p did not, however, appear to be enough to induce autophagy. We cannot rule out the possibility that miR-342-5p can directly target autophagy-related components, but we identified, for the first time, miR-342-5p as an autophagy-regulating miRNA.

In a previous study on SW1353, OUMS-27, CH2879 and L835 cells, inhibition of Bcl-xL and Bcl-2 with ABT-737 restored the chemosensitivity to doxorubicin and CDDP (5 µM), with the more marked inhibition in cell viability after 72 h [15]. This study also used 10 µM CDDP in combination with WEHI-539, a selective inhibitor of Bcl-xL, to induce apoptosis of chondrosarcoma cells after 72 h of treatment [25]. In contrast, in our study, miR-491-5p, which inhibits Bcl-xL, and miR-342-5p, which downregulates both Bcl-2 and Bcl-xL, did not chemosensitize chondrosarcoma cells to CDDP. We used sublethal doses of CDDP to induce cell cycle stalling at the S and/or G2/M phases (0.33 µM for OUMS-27, 1.65 µM for L835 and CH2879 and 3.3 µM for SW1353). We performed functional analysis 24 h after CDDP treatment. Therefore, the chosen kinetics and these sub-lethal doses of CDDP may not be adequate to reveal any chemosensitizing effect of these miRNAs. Other experiments are required to explore this hypothesis.

We showed that miR-491-5p and miR-342-5p do not affect the healthy primary HAC cell cycle, nor do they cause any cell death, indicating their biosafety on non-cancerous chondrocytes. Finally, we investigated their effects on a 3D organoid chondrosarcoma model that mimics the in vivo microenvironment, rather than using a classic xenograft model. We previously used collagen sponge scaffolds to successfully re-differentiate dedifferentiated HACs, or to differentiate mesenchymal stem cells into chondrocytes, with the combination of growth factors/siRNAs/hypoxia [53,54,55]. These collagen sponges scaffolds have also been used to study the impact of irradiations in a 3D chondrosarcoma model [56]. As previously described [56], this 3D organoid chondrosarcoma model mimics an intermediated grade chondrosarcoma tissue cellularity with a homogeneous distribution of SW1353 cells into the 3D scaffold. A higher Ki67 proliferation index (33% ± 4%), measured by immunochemistry staining, was also determined after 7 days of culture of SW1353 cells into the collagen scaffold [56]. Compared with the conventional subcutaneous xenograft implantation in *nude* mice, our 3D culture model makes it possible to control oxygen tension during experimentations. The effects of the miRNAs were thus studied under hypoxia to closely mimic the in situ microenvironment of chondrosarcomas. In this model, we found that miR-491-5p was not able to induce cell death, whereas miR-342-5p provoked cell death in normoxia and hypoxia, with a higher level of significance in the more biologically relevant hypoxia. This miRNA would therefore be the most effective if considering miRNAs as a therapeutic avenue for the treatment of chondrosarcomas.

## 4. Materials and Methods

### 4.1. Cell Culture

The chondrosarcoma cell line SW1353 (ATCC^®^ HTB-94) was grown and treated in high glucose-Dulbecco’s modified Eagle’s medium (HG-DMEM, Biowest, Nuaillé, France) supplemented with 10% fetal calf serum (FCS) (Eurobio Scientific, Courtaboeuf, France), 3 µg/mL ciprofloxacin (Sigma-Aldrich, Saint-Louis, MO, USA) and 0.5 µg/mL amphotericin (Eurobio Scientific, Courtaboeuf, France). The chondrosarcoma cell lines OUMS-27, L835 and CH2879 were kindly provided by Y. Saintigny (LARIA, Caen, France) and they initially originated from J.V.M.G Bovée’s laboratory (Department of Pathology, Leiden, the Netherlands). They were grown and treated in RPMI 1640 medium (Eurobio Scientific, Courtaboeuf, France) with 10% FCS and a mixture of penicillin (100 IU/mL) and streptomycin (100 µg/mL) (Eurobio Scientific, Courtaboeuf, France).

Human articular chondrocytes (HACs), obtained with appropriate ethical approval, were prepared from macroscopically healthy zones of femoral heads obtained from patients undergoing joint arthroplasty, as previously described [57]. The study was performed in full accordance with local ethics committee guidelines and all the cartilage samples were collected after written and informed consent of the donors according to French legislation. All the experimental protocols were approved by the French Ministry of Higher Education and Research (Ethics Committee for Research on Human Samples CODECOH: DC 2014–2325). Chondrocytes were seeded at 4 × 10^4^ cells/cm^2^ in plastic dishes, with a medium consisting of HG-DMEM supplemented with 10% FCS and a mixture of 100 IU/mL penicillin, 100 mg/mL erythromycin and 0.25 mg/mL fungizone (Eurobio Scientific, Courtaboeuf, France).

All cells were certified mycoplasma-free with PCR analysis. They were maintained in a humidified atmosphere containing 5% CO_2_ at 37 °C. Treatments were performed under normoxia (21% O_2_) and hypoxia (3% O_2_). Hypoxic cultures, including any handling, were exclusively performed in a sealed chamber, with a controlled rate of oxygenation [57].

For 3D experiments, SW1353 cells were grown in collagen scaffolds manufactured by Symatèse Biomatériaux (Chaponost, France), as described previously for the redifferentiation of HACs [53] or to study the impact of radiation in a 3D chondrosarcoma model [56] with some modifications. The collagen sponge scaffolds were composed of native type I collagen (90–95%) and type III collagen (5–10%) from calf skin. These sponges, 2 mm thick and 5 mm in diameter, were cross-linked with glutaraldehyde to increase their stability and sterilized using β-radiation. Briefly, SW1353 cells were seeded at 2x10^5^ cells/sponge in 96-well culture plates and incubated at 37 °C and 5% CO_2_ in HG-DMEM supplemented with 10% FCS and antibiotics. After 1 h, each cell construct was transferred to 24-well plates and incubated in the same medium pre-equilibrated with 3% O_2_ by bubbling for culture under hypoxia or without O_2_ pre-equilibration for culture under normoxia.

### 4.2. Drug and miRNAs

Cisplatin (Cis-diaminedichloroplatinum or CDDP) was purchased from Mylan (Merck Santé SAS, Lyon, France). Sublethal doses of CDDP were used during the transfection of miRNA as follows: 1 µg/mL (3.3 µM) for SW1353, 0.5 µg/mL (1.65 µM) for L835 and CH2879 cells and 0.1 µg/mL (3.3 µM) for OUMS-27 cells. All miRNA mimics were purchased from Dharmacon (Horizon Discovery, Cambridge, UK). MiRNA-Control (miR-Ctrl, MIMAT0000039), was based on a *C. elegans* miRNA sequence that has been confirmed to have minimal sequence identify with human miRNAs. Human miRNAs hsa-miR-491-5p (MIMAT0002807, noted miR-491), hsa-miR-342-5p (miR-342, MIMAT0004694), hsa-miR-541-5p (miR-541, MIMAT0004919), hsa-miR-625-5p (miR-625, MIMAT0003294) and hsa-miR-149-5p (miR-149, MIMAT0000450) were also used in this study. MiRNA hairpin inhibitors were purchased from Dharmacon: hsa-miRNA-491-5p-hairpin inhibitor (anti-miR-491, IH-300751-06), hsa-miRNA-342-5p-hairpin inhibitor (anti-miR-342, IH-301083-02) and miRNA hairpin inhibitor Negative Control (anti-miR-ctrl, IN-001005-01).

### 4.3. Real-Time Cell Analysis (xCELLigence)

MiRNA- and CDDP-mediated cytotoxicity were initially monitored using high-throughput screening with the Real-Time Cell Analyzer (RTCA) multi-plate instrument (xCELLigence System; ACEA, Ozyme, Saint-Quentin-en-Yvelines, France) under normoxia. This system monitors cellular events in real-time by measuring electrical impedance across interdigitated micro-electrodes integrated into the bottom surfaces of 96-well E-plates VIEW (Ozyme, Saint-Quentin-en-Yvelines, Trapp, France). RTCA 2.1.0 software calculates the cell index (CI) based on impedance. CI correlates with the area of cells attached to the bottom of the plate as described elsewhere [58]. Briefly, 3.5 × 10^3^ SW1353 cells/well were plated in 96-well E-plates VIEW. They were left to grow for 24 h before transfection with 20 nM miRNAs using Interferin^TM^ (Polyplus-Transfection, Strasbourg, France). In each plate, miR-Ctrl was used as a negative control of cytotoxicity. The cells were treated with or without CDDP 24 h post-transfection. Impedance was continuously measured until the end of the treatment (i.e., 96 h post-transfection and 120 h after seeding). MiRNA effect was established according to three criteria: the shape of the curve, the area under the curve (AUC) and the CI at the end of the experiment. Standard deviations of triplicates were analyzed with the RTCA software. Endpoint morphological analysis of cells was also done in a high-throughput cell imaging system (Cellavista; Roche, Basel, Switzerland) at the end of the experiment.

### 4.4. Transfection of miRNA and CDDP Treatment

For individual characterization and validation studies, exponentially growing cells were seeded at 7.5 × 10^4^ cells/cm^2^ (for OUMS-27), 1 × 10^4^ cells/cm^2^ (for SW1353 cells) and 2 × 10^4^ cells/cm^2^ (for L835 and CH2879 cells). Cell lines were transfected 24 h after seeding with 20 nM of miRNA mimics using INTERFERin^TM^ (Polyplus-Transfection, Strasbourg, France) according to the manufacturer’s instructions. An additional CDDP treatment was realized 48 h post-transfection with sublethal doses of CDDP.

For 3D experiments, SW1353 cells were transfected with 20 or 50 nM miRNA the day after seeding in the collagen sponge. HACs were transfected with 20 nM miRNA at passage 0% and at 80% confluency. In all cases, cells and media were harvested at the end of the experiment (72 h post-transfection) for further analysis.

### 4.5. Metabolic Activity Analysis

SW1353 cells were seeded onto 96-well microplates at a density of 3.5 × 10^3^ cells/well in triplicate. Transfection of 20 nM miRNA was performed 24 h later with or without an additional CDDP treatment 48 h post-transfection, as described above. Cellular metabolic activity was estimated 72 h post-transfection using the XTT assay (Roche, Basel, Switzerland). This assay is based on the cleavage of the tetrazolium salt XTT to a soluble formazan salt only in viable cells. Therefore, the amount of formazan dye formed is directly correlated with the number of metabolically active cells in the culture. The medium was removed and the cells were incubated with XTT working solution (per well: 100 μL of culture medium, 50 μL of XTT and 1 μL of electron coupling reagent). Optical density (OD) was measured at 450 nm and 600 nm with an absorbance microplate reader (Spark 10M; Tecan, Lyon, France) after 1 h of incubation.

### 4.6. Cytotoxicity Assay

In parallel with the metabolic activity analysis, a bioluminescence cytotoxicity assay kit (Interchim, Montluçon, France) was used to evaluate miRNA-induced cytotoxicity 72 h post-transfection. This kit is based on the measurement of adenylate kinase (AK) rapidly released into the culture medium upon damage to the plasma membrane. AK detection was carried out in the supernatant in a simple one-step procedure involving two chemical reactions. The first reaction converts ADP to ATP by AK. The second reaction uses luciferase to catalyze the formation of the light from ATP and luciferin. The emitted light was then measured with a luminescence microplate reader (Spark 10M, Tecan Lyon, France) and expressed as relative luciferase units (RLU), reflecting the number of dead cells.

### 4.7. Cell Cycle Analysis

Adherent cells were harvested by trypsinization. The supernatant, containing the detached cells, and the trypsinized cells were pooled, centrifuged (1400 rpm, 10 min), rinsed with PBS, fixed in 70% ethanol, and stored at −20 °C until analysis. Fixed cells were then centrifuged (3000 rpm, 10 min) and rinsed with PBS to remove all traces of ethanol. After centrifugation (2000 rpm, 5 min), the cells were resuspended and incubated for 30 min in a PBS solution containing 20 µg/mL propidium iodide (Sigma-Aldrich, Saint-Louis, MO, USA) and 100 µg/mL RNase (Fisher Scientifics SAS, Illkirch, France). Cell cycle phase distribution was analyzed by flow cytometry using the Cytoflex S Flow Cytometer (Beckman Coulter France SAS, Paris, France). Data were computed with the Cytexpert^®^ acquisition software (Beckman Coulter France SAS, Paris, France).

### 4.8. Analysis of Nuclear Morphology

Both detached and adherent cells were pooled and applied to a poly-L-lysine-coated glass slide by cyto-centrifugation at 850 rpm for 5 min (Cytospin, Fisher Scientifics SAS, Illkirch, France). They were fixed with a solution of ethanol/chloroform/acetic acid (6:3:1) before DNA staining. Slides were then treated with the UltraCruz™ Mounting Medium with DAPI (4′,6-diamidino-2-phenylindole, dilactate) (Santa Cruz Biotechnology, Dallas, TX, USA). The observations were carried out under a fluorescence microscope (Nikon Eclipse Ti; Nikon Instruments Inc., Melville, NY, USA).

### 4.9. Western Blotting

Both detached and adherent cells were centrifuged at 1400 rpm for 5 min, washed with PBS and stored at −20 °C until analysis. Total proteins were extracted using the RIPA-lysis buffer as previously described [59]. Protein concentration was assessed according to the Bradford colorimetric procedure (Biorad, Hercules, CA, USA). Then, 15 µg of total proteins were separated in 10% or 12% polyacrylamide gels containing 0.1% SDS and transferred to a polyvinylidene difluoride membrane (PVDF, Biorad, Hercules, CA, USA) using the Trans Blot^®^Turbo^TM^ Transfer system (BioRad, Hercules, CA, USA). Unspecific binding sites of the membranes were blocked with 10% non-fat milk powder in Tris-buffered saline with 0.1% Tween (TBST) for 1 h. Then, protein levels were analyzed by immunoblotting with antibodies from Cell Signaling Technology (Ozyme, Saint-Quentin-en-Yvelines, France), according to the manufacturer’s instructions: EGFR (4267), PARP (9542), Caspase-9 (9502), Bcl-xL (2764), Cyclin D1 (2878), Bak (3814), phospho-p44/42 MAPK (Thr202/Tyr204 of Erk1/2, 4370), p44/42 MAPK (Erk1/2, 9102), phospho-AKT (Ser473, 4060), AKT (9272). We also used McL-1 (S-19), GAPDH (FL-335) and β-Tubulin (D-10) antibodies from Santa Cruz Biotechnology (Dallas, TX, USA), and the Bcl-2 (M0887) antibody from DAKO. After incubation, membranes were washed in TBST, followed by an incubation with HRP-conjugated goat anti-rabbit or mouse IgG antibody (Jackson Immunoresearch, West Grove, PA, USA). Signals were visualized with a chemiluminescence imaging system (ChemiDoc^TM^ Touch imaging system; Biorad, Hercules, CA, USA). Each immunoblot is representative of at least three distinct experiments. Protein expression was estimated by quantifying the density of immunoblots bands adjusted to β-Tubulin or GAPDH (ImageLab^®^ software; Biorad, Hercules, CA, USA).

### 4.10. Real-Time Detection of Caspase-3/7 Mediated Apoptosis

Caspase-3/7 activity was assessed using the Incucyte^®^ caspase-3/7 green apoptosis assay reagent (Essen BioScience, Ltd., Royston, UK). This reagent couples the activated caspase-3/7 recognition motif (DEVD) to NucView™488, a DNA intercalating dye. Thus, caspase-3/7 activity correlates with an increase in the green fluorescence in the nuclei. Briefly, 3.5 × 10^3^ SW1353 cells/well were grown in 96-well plates and monitored in the Incucyte^®^ S3 acquiring images (objective × 10) every 3 h in two separate regions per well after transfection of 20 nM miRNA. Each experiment was done in triplicate and analyzed using Incucyte^®^ software.

### 4.11. RNA Isolation and PCR Assay

Total RNA (including miRNA) was isolated from transfected cells using the NucleoSpin^®^ miRNA kit (Macherey-Nagel SAS, Herdt, France) according to the manufacturer’s instructions. RNA quantity and quality were assessed using the NanoDrop^TM^ 2000 spectrophotometer (ThermoFisher Scientific, Waltham, MA, USA). Reverse transcription was performed to generate cDNA from 1 µg of total RNA with the miScript II RT kit (Qiagen SAS, Courtaboeuf, France). PCR was performed on a CFX96 Touch real Time PCR (Biorad, Hercules, CA, USA). Data were analyzed using CFX manager software. For the detection of miRNAs, we used QuantiTect™ SYBR Green PCR master mix (Qiagen SAS, Courtaboeuf, France) with an universal miScript primer and specific miScript primers provided by Qiagen: Hs_miR-491-5p_1 (miR-491-5p), Hs_miR-342-5p_1 (miR-342-5p), Hs_miR-541*_1 (miR-541-5p), Hs_miR-625_3 (miR-625-5p), Hs_miR-149_1 (miR-149-5p), Hs_RNU6-2_11 (U6 small nuclear 6 or RNU-6B) and Hs_miR-15a_1 (miR-15a). The cycling conditions were 15 min for polymerase activation at 95 °C, followed by 40 cycles at 94 °C for 15 s, 55 °C for 30 s and 70 °C for 30 s. In addition, melting curves were performed to ensure specificity of PCR products. Expression values of miRNAs were normalized to RNU-6B and to miR-15a with CFX manager software. They were expressed as mean of triplicate samples ± SD.

For RNA transcript quantification, we used GoTaq^®^qPCR Master Mix (Promega, Charbonnières-les-Bains, France). The following primers were purchased from Eurogentec SA (Angers, France): EGFR F-5′-TGGCATCTTTAAGGGCTCCA-3′, EGFR R-5′-TGGCTAGTCGGTGTAAACGT-3′, PPIA1 F-5′-CGGATTTGATCATTTGGTG-3′, PPIA1 R-5′-CAGGGAATACGTAACCAG-3′, GAPDH F-5′-CCTGCACCACCAACTGCTTA-3′, GAPDH R-5′-GGCCAT CCACAGTCTTCTGGG-3′. GAPDH and PPIA1 were both used as endogenous reference genes to normalize EGFR expression, expressed as the mean of triplicate samples ± SD.

### 4.12. Luciferase miRNA Target Reporter Assay

Targetscan 7.2 2020 (http://www.targetscan.org, accessed on 24 May 2021) was used for miRNA target prediction. Wild type (WT) and mutant (MUT) vectors of BCL2L1-3′UTR and BCL2-3′UTR were purchased from GeneCopoeia (Rockville, MD, USA). The full length BCL2L1-3’UTR (217HmiT108616-MT05; 1489 bp) and of the proximal part of BCL2-3’UTR (217HmiT016211a; 2761 bp) were inserted downstream of a Gaussia luciferase (GLUC) reporter gene in the pEZMX-MT05 vector. This vector also carries the secreted alkaline phosphatase (SEAP) reporter gene for normalization of GLUC luciferase activity, as a function of transfection efficiency. SW1353 cells were seeded at 8 × 10^4^ cells/well (triplicates) in a 24-well plate the day before transfection. The cells were co-transfected with 20 nM miRNA mimic or miRNA hairpin inhibitor and with BCL2-3′UTR vector (0.25 ng/µL) using jetOPTIMUS^TM^ DNA transfection reagent (Polyplus-Transfection, Strasbourg, France). BCL2L1-3′UTR vector (1 ng/µL) and miRNA were co-transfected with EndoFectin transfection reagent (Genecopoeia, Rockville, MD, USA). The culture medium was collected 24 h later to measure luciferase activities with the secrete-pair dual luminescence assay kit (Genecopoeia, Rockville, MD, USA) and a luminescence microplate reader (Spark 10M, Tecan, Lyon, France). Luciferase activities were expressed as relative luciferase units (RLU) corresponding to GLUC/SEAP ratio.

### 4.13. Autophagy Assay

Cell Meter^TM^ autophagy fluorescence imaging kit (AAT Bioquest^®^, Sunnyvale, CA, USA) was used to analyze the autophagic activity of the cells. The kit uses the Autophagy Green^TM^ reagent as a specific autophagosome marker. SW1353 cells were seeded onto 96-well microplates at a density of 3.5 × 10^3^ cells/well in triplicates. They were transfected 24 h later with 20 nM miRNA as described above, and incubated for 72 h. As positive controls, cells were incubated for the last 24 h in PBS with 5% FCS. Seventy-two hours post-transfection, the medium was replaced by the Autophagy Green^TM^ working solution. The cells were incubated in a 37 °C, 5% CO_2_ incubator for 1 h. After washing with PBS, the nuclei were counterstained with DAPI (Santa Cruz Biotechnology, Dallas, TX, USA). The cells were then observed under a fluorescence microscope (Nikon Eclipse Ti; Nikon Instruments Inc, Melville, NY, USA). Autophagosomes and nuclei were counted with ImageJ software (National Institutes of Health, Bethesda, MD, USA).

### 4.14. Statistical Analysis

All experiments were repeated at least three times. Values are reported as means ± SD. Statistical significances of the mean of at least three independent experiments were assessed using one-way ANOVA corrected for multiple comparisons using Dunnett’s test, or two-way ANOVA corrected for multiple comparisons using Sidak’s test. Alternatively, two-tailed unpaired student’s *t*-test with Welch’s correction was used to analyze statistical differences within representative experiments performed in triplicate. Statistical analyses were carried out using GraphPad Prism 7 software (San Diego, CA, USA). *p*-values of less than 0.05 were considered significant: *** *p* < 0.001, ** *p* < 0.01, and * *p* < 0.05.

## 5. Conclusions

Our study unambiguously demonstrated the antiproliferative, antimetabolic and cytotoxic effects of miR-491-5p and miR-342-5p on the SW1353 chondrosarcoma cell line. However, neither miRNA was able to induce chemosensitivity to CDDP in our experimental conditions, but the tumor suppressive effects of these miRNAs were validated in three out of four chondrosarcoma cell lines cultured under normoxia and hypoxia. MiR-491-5p inhibited the expression of EGFR, whose constitutive activation is associated with cell proliferation, survival and migration. Both miRNAs induced apoptosis and miR-342-5p also promoted autophagy. We identified Bcl-xL and Bcl-2 as direct targets of miR-342-5p, and Bcl-xL as a direct target of miR-491-5p in chondrosarcoma chondrocytes. This study therefore provides additional evidence of the therapeutic potential to specifically target Bcl-2 family members in chondrosarcomas. Only miR-342-5p appeared to be effective in a more relevant 3D culture model in hypoxia. The loss of efficiency of miR-491-5p in the organoid culture system demonstrates the importance of considering the tumor microenvironment in the development of new efficient therapeutic tools.

## Data Availability

The data presented in this study are available in Supplementary Materials.

## Supplementary Materials

The following are available online at https://www.mdpi.com/article/10.3390/ijms22115590/s1, Figure S1: Identification of miRNAs with cytotoxic and chemosensitizing effects using a high-throughput cell imaging system. Figure S2: Determination of miRNA transfection efficiency in SW1353 cells. Figure S3: Analysis of chemosensitizing effects of miRNA-491 and miRNA-342 on SW1353 cells. Figure S4: Effects of miR-491-5p and miR-342-5p on OUMS-27 and CH2879 cells. Figure S5: Effects of miR-491-5p and miR-342-5p on L835 chondrosarcoma cells and on HACs. Figure S6: Analysis of the chemosensitizing effects of miR-491-5p and miR-342-5p on several chondrosarcoma cell lines. Figure S7: Analysis of some apoptotic/antiapoptotic proteins in OUMS-27 and CH2879 cells. Figure S8: Study of some survival/death proteins and mitogenic signaling pathways in OUMS-27 and CH2879 cells. Figure S9: Effects of miR-491-5p and miR-342-5p on SW1353 cells cultured in 3D. Table S1: Predicted and direct targets of miR-491-5p. Table S2: Predicted and direct targets of miR-342-5p.

## Abbreviations

AKT: Protein kinase B; Bak: Bcl-2 homologous antagonist killer; Bax: Bcl-2-associated X; Bcl-2: B-cell lymphoma-2; Bcl-xL: Bcl-2 lymphoma-extra large; CDDP: Cis-diaminedichloroplatinum or cisplatin; EGFR: Epidermal Growth Factor Receptor; ERK: Extracellular signal-Regulated Kinase; HAC: Human Articular Chondrocytes; Mcl-1: Myeloid cell leukemia-1; miR: MicroRNA or miRNA; UTR: Untranslated Region.
