# Supplementary material for: Tumor Suppressive Role of miR-342-5p in Human Chondrosarcoma Cells and 3D Organoids
Source: Int J Mol Sci. 2021 May 25;22(11):5590. doi: 10.3390/ijms22115590 (PMC8197525; doi:10.3390/ijms22115590)
Supplement: Supplementary file 1 [file ijms-22-05590-s001.zip › Supp data S1 a╠Ç S9-TablesS1-S2 ijms 05-23-2021.pdf]

Tumor suppressive role of miR-342-5p in human chondrosarcoma cells and 3D organoids

Clément Veys[1], Abderrahim Benmoussa[1,2], Romain Contentin[1], Amandine Duchemin[1], Emilie Brotin[3,4,5], Jérôme Lafont[6], Yannick Saintigny[7,8], Laurent Poulain[4,5], Christophe Denoyelle[3,4,5], Magali Demoor[1], Florence Legendre[1] and Philippe Galéra[1]

[1] Normandie Univ, UNICAEN, BIOTARGEN, 14000, Caen, France.
[2] Research Center of the UHC Sainte-Justine and Department of Nutrition, Université de Montréal, Montréal, Québec, Canada, H3T 1C54.
[3] Normandie Univ, UNICAEN, ImpedanCELL Platform, Federative Structure 4206 ICORE, 14000, Caen, France.
[4] Normandie Univ, UNICAEN, INSERM U1086 ANTICIPE, Biology and Innovative Therapeutics for Ovarian Cancer (BioTICLA), 14000, Caen, France.
[5] UNICANCER, Comprehensive Cancer Center F. Baclesse, 14000, Caen, France.
[6] CNRS UMR 5305, Laboratory of Tissue Biology and Therapeutic Engineering, Université Claude Bernard Lyon 1, Univ Lyon, 69367, Lyon, France.
[7] LARIA, iRCM, François Jacob Institute, DRF-CEA, 14000 Caen, France.
[8] Normandie Univ, ENSICAEN, UNICAEN, CEA, CNRS, UMR6252 CIMAP, 14000, Caen, France.

Correspondence: Philippe Galéra ; philippe.galera@unicaen.fr

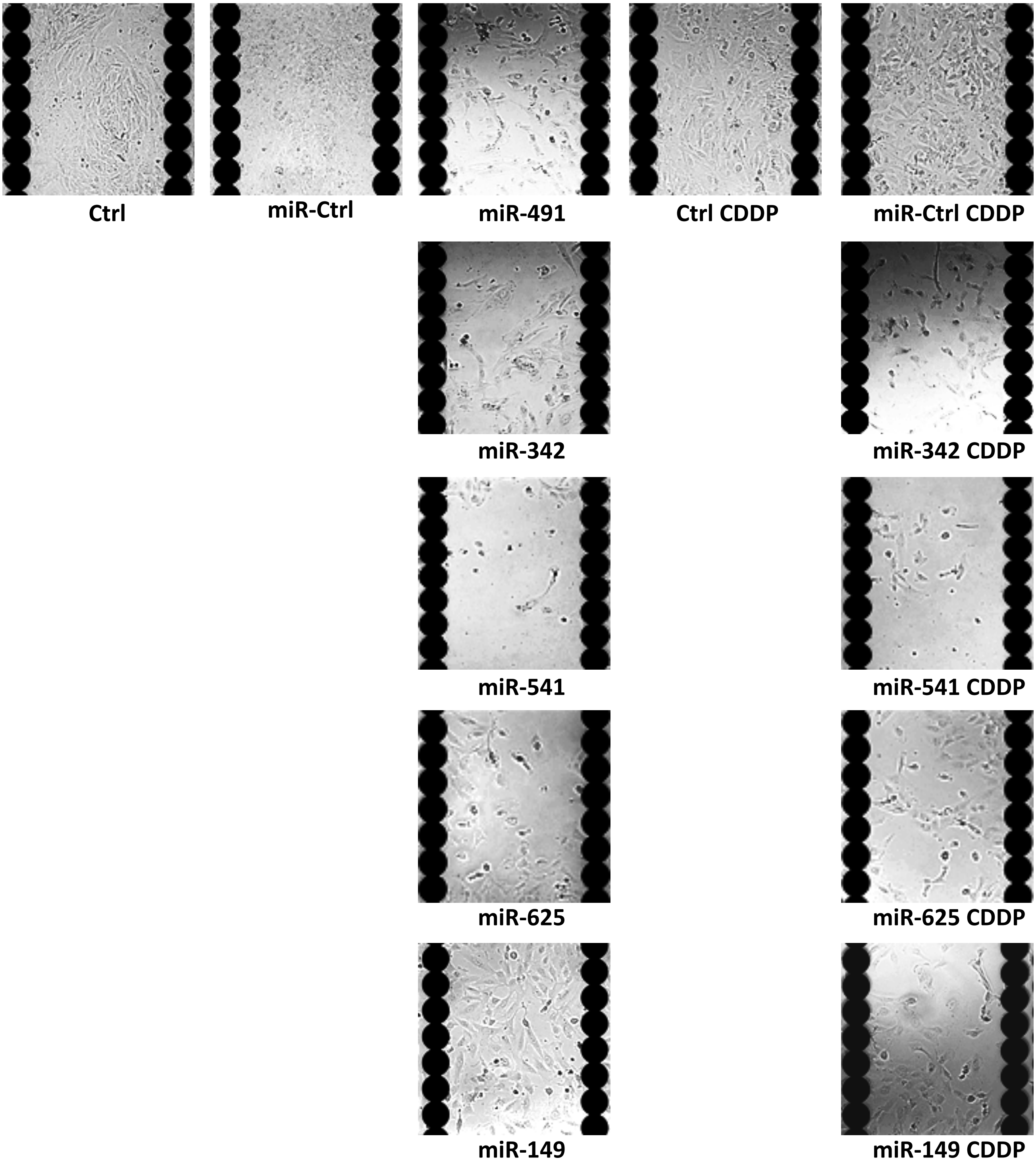

Supplementary Figure S1: Identification of miRNAs with cytotoxic and chemosensitizing effects using a high-throughput cell imaging system. SW1353 cells were seeded onto 96-well E-plates VIEW, and treated with miRNA and cisplatin (CDDP) as described in Figure 1. Endpoint morphological analysis of the cells was done 120 h after seeding with the Cellavista imaging system. MiR-491-5p was used as a positive control of cytotoxicity.

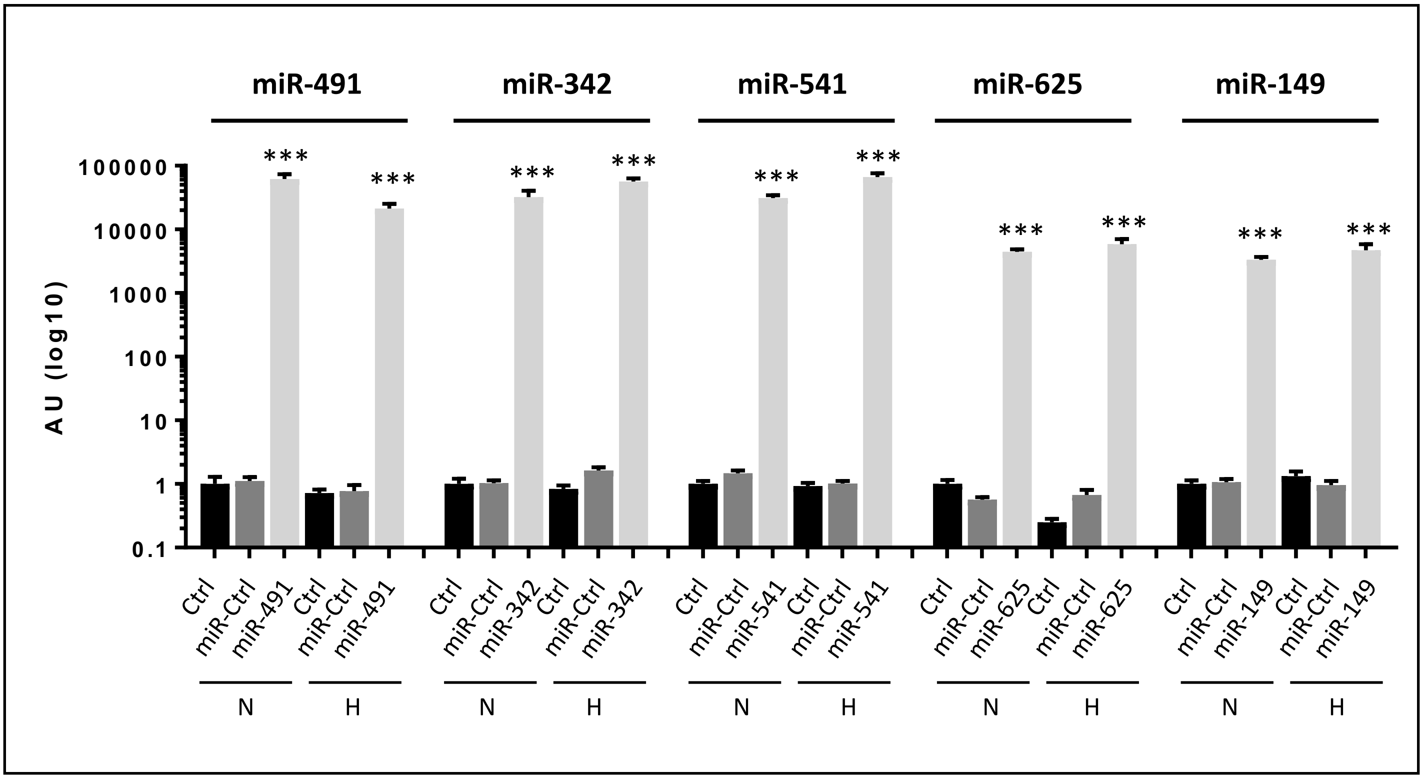

Supplementary Figure S2: Determination of miRNA transfection efficiency in SW1353 cells. SW1353 cells were cultured and transfected with miRNA, under normoxia (N) or hypoxia (H), as described in Figure 3. Total RNA was extracted 72 h post-transfection as described in the Materials and Methods section. Relative expressions of *miR-491-5p, miR-342-5p, miR-541-5p, miR-625-5p and miR-149-5p* were determined by RT-quantitative PCR and normalized to both *RNU-6B* and *miR-15a* levels. The results are representative of three independent experiments. Data are expressed as mean of triplicate samples ± SD in arbitrary units (AU) in log scale. The significance of the results between miR-Ctrl and miRNA-treated cells was assessed using the Student's *t*-test (***: p<0.001).

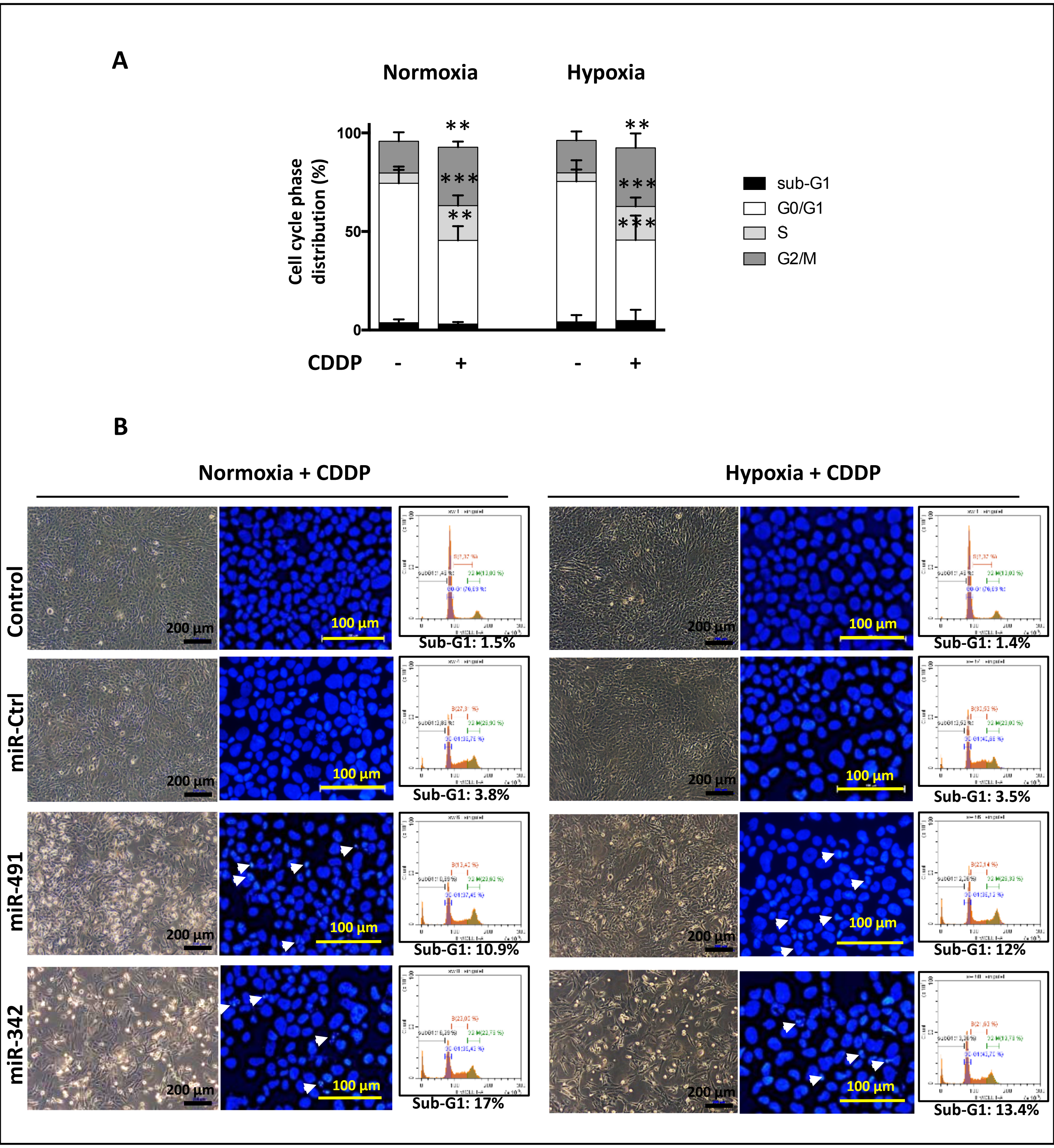

Supplementary Figure S3: Analysis of chemosensitizing effects of miRNA-491 and miRNA-342 on SW1353 cells. SW1353 cells were cultured and transfected with miRNA as described in Figure 3. Analyses were carried-out 72 h post-transfection. (A) Cell cycle phase distribution was analyzed using flow cytometry. The histogram shows the analysis of five independent experiments (mean ± SD) with the different phases of the cycle for non-transfected cells (Ctrl). Statistically significant differences in the percentage of events in the sub-G1, G0/G1, S and G2/M phases, between Ctrl and CDDP-treated cells were determined using one-way ANOVA (**: $p<0.01$, ***: $p<0.001$). (B) In the left panels, cell morphology was obtained using photonic microscopy at the end of the experiment performed in normoxia or hypoxia. In the middle panels, nuclear morphology was obtained after DAPI staining as described in the Materials and Methods section. White arrowheads show cells with condensed and/or fragmented chromatin and cellular debris. In the right panels, DNA content histograms were obtained using flow cytometry. Images shown are representative of five independent experiments.

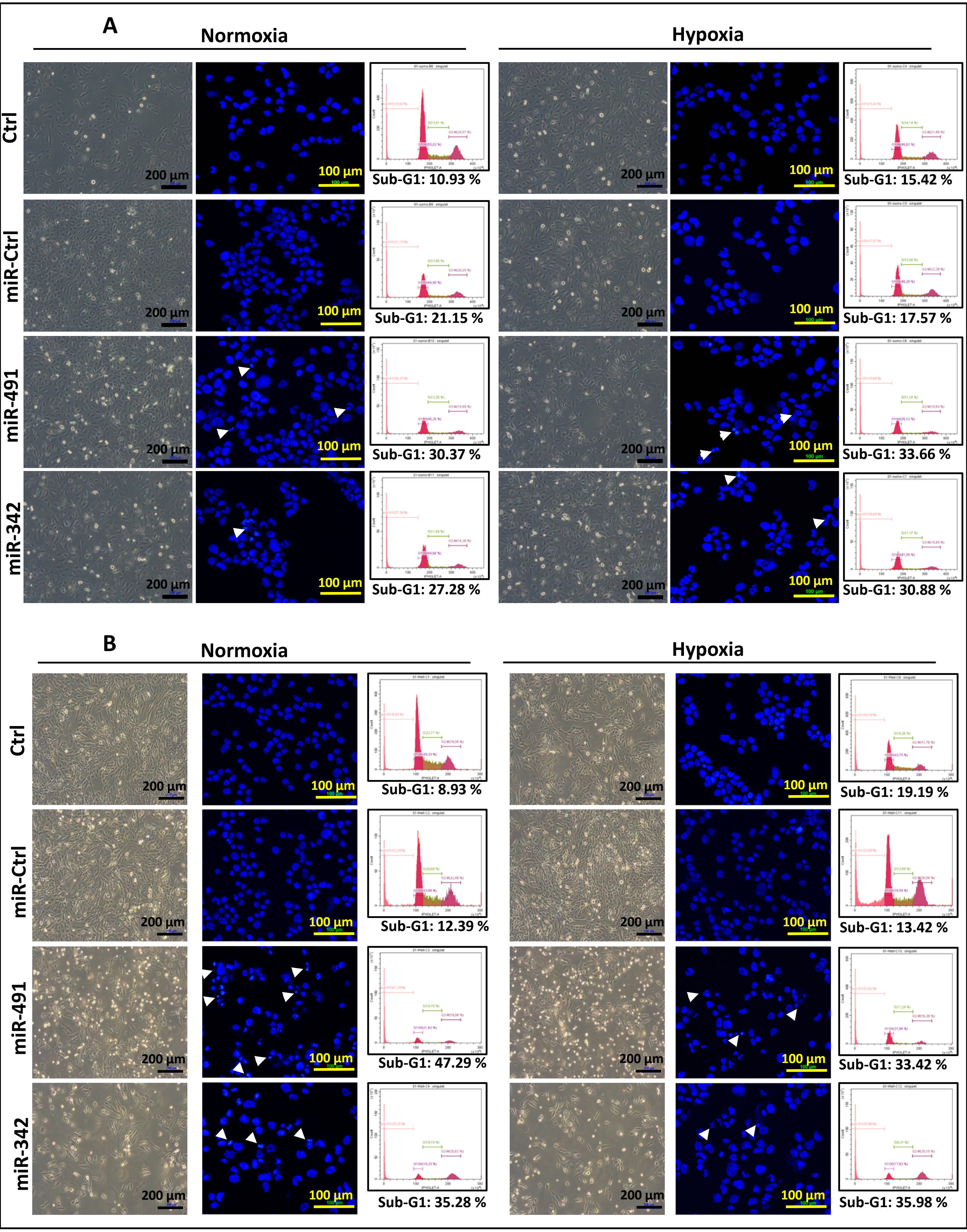

Supplementary Figure S4: Effects of miR-491-5p and miR-342-5p on OUMS-27 and CH2879 cells. OUMS-27 cells (A) and CH2879 cells (B) were cultured and transfected with miRNA under normoxia or hypoxia as described in Figure 4. Analyses were carried out 72 h post-transfection. In the left part of each panel, cell morphology was obtained using photonic microscopy at the end of the experiment. In the middle panels, nuclear morphology was obtained after DAPI staining. White arrowheads show cells with condensed and/or fragmented chromatin and cellular debris. In the right panels, DNA content histograms were obtained using flow cytometry. Images shown are representative of at least four independent experiments.

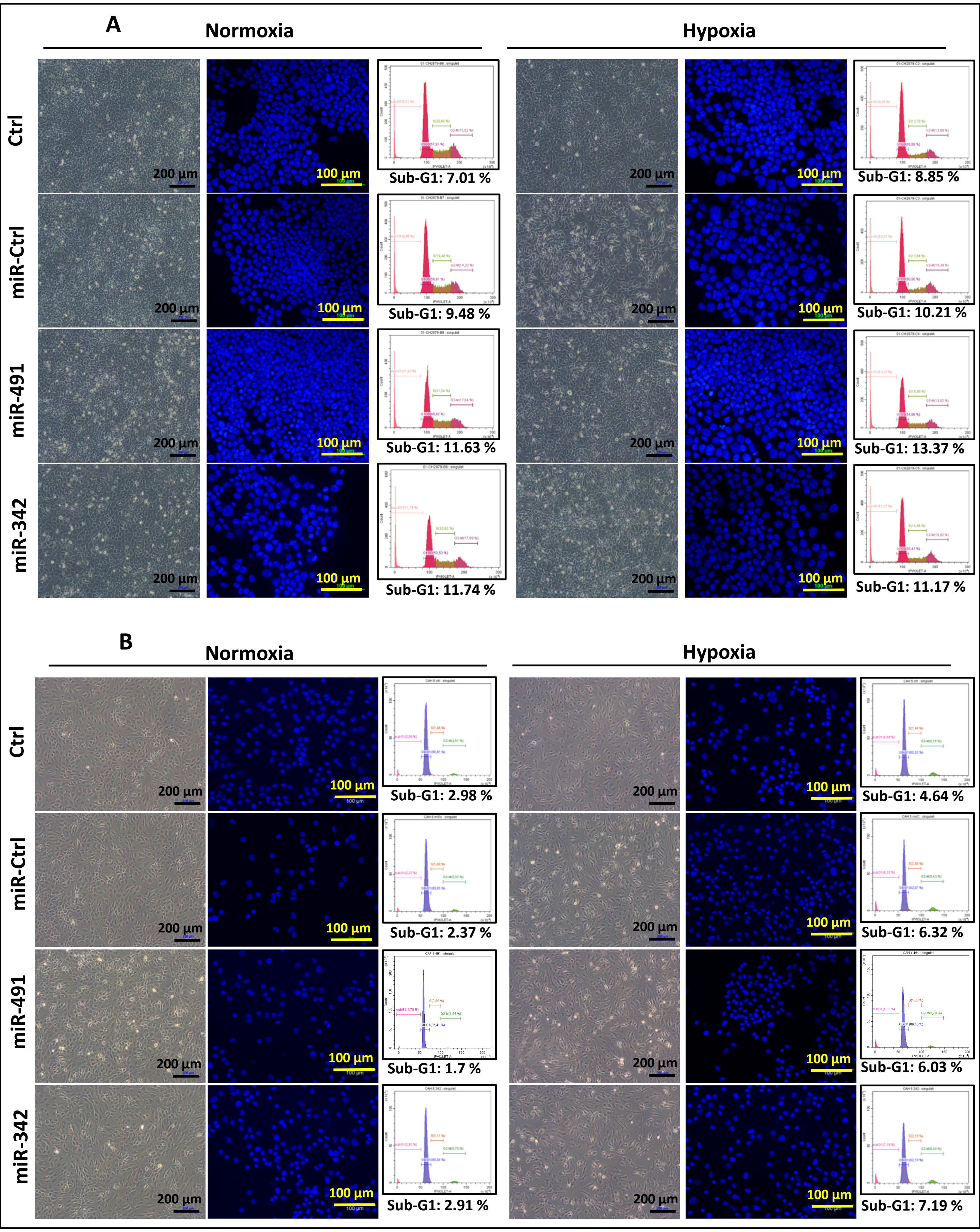

Supplementary Figure S5: Effects of miR-491-5p and miR-342-5p on L835 chondrosarcoma cells and on HACs. L835 cells (A) and HACs (B) were cultured and transfected with miRNA in normoxia or hypoxia as described in Figure 4. Analyses were carried out 72 h post-transfection. In the left panels, cell morphology was obtained using photonic microscopy at the end of the experiment. No condensed and/or fragmented chromatin or cellular debris was detected. In the middle panels, nuclear morphology was obtained after DAPI staining. In the right panels, DNA content histograms were obtained using flow cytometry. Images shown are representative of at least four independent experiments.

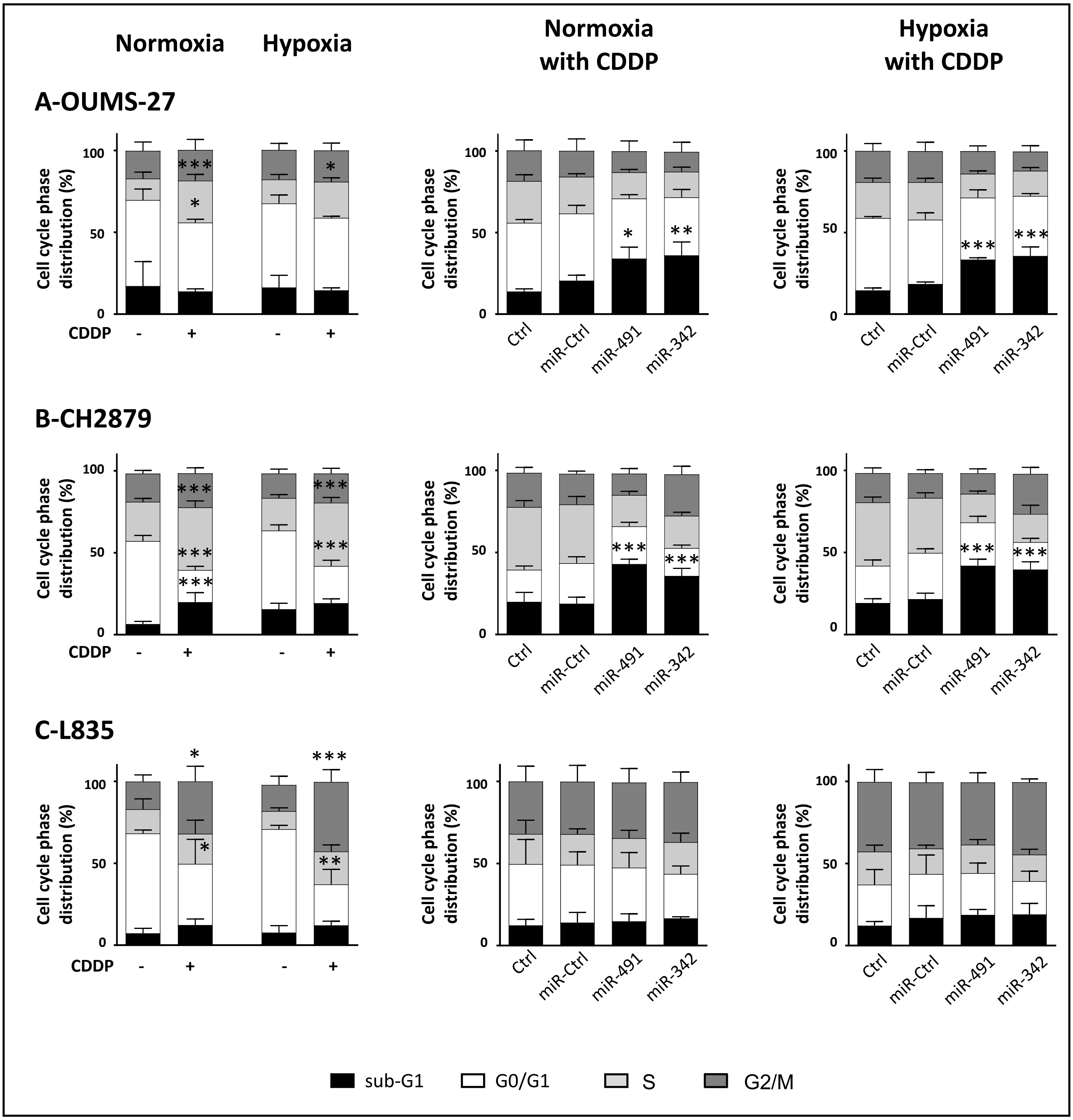

Supplementary Figure S6: Analysis of the chemosensitizing effects of miR-491-5p and miR-342-5p on several chondrosarcoma cell lines. OUMS-27 cells (A), CH2879 cells (B) and L835 cells (C) were cultured and transfected with miRNA as described in Figure 4. For each chondrosarcoma cell line, treatments with cisplatin (CDDP) were performed 48 h post-transfection. Cell cycle phase distribution was analyzed using flow cytometry 72 h post-transfection. The histograms represent the analysis of the different phases of the cell cycle of at least four independent experiments (mean ± SD). Left panels: Statistically significant differences in the percentage of events in the sub-G1, G0/G1, S and G2/M phases, between Ctrl and CDDP-treated cells were determined using one-way ANOVA. Middle and right panels: Statistically significant differences in the percentage of sub-G1 events between miR-Ctrl and miRNA-treated cells were determined using one-way ANOVA (*: $p<0.05$, **: $p<0.01$, ***: $p<0.001$).

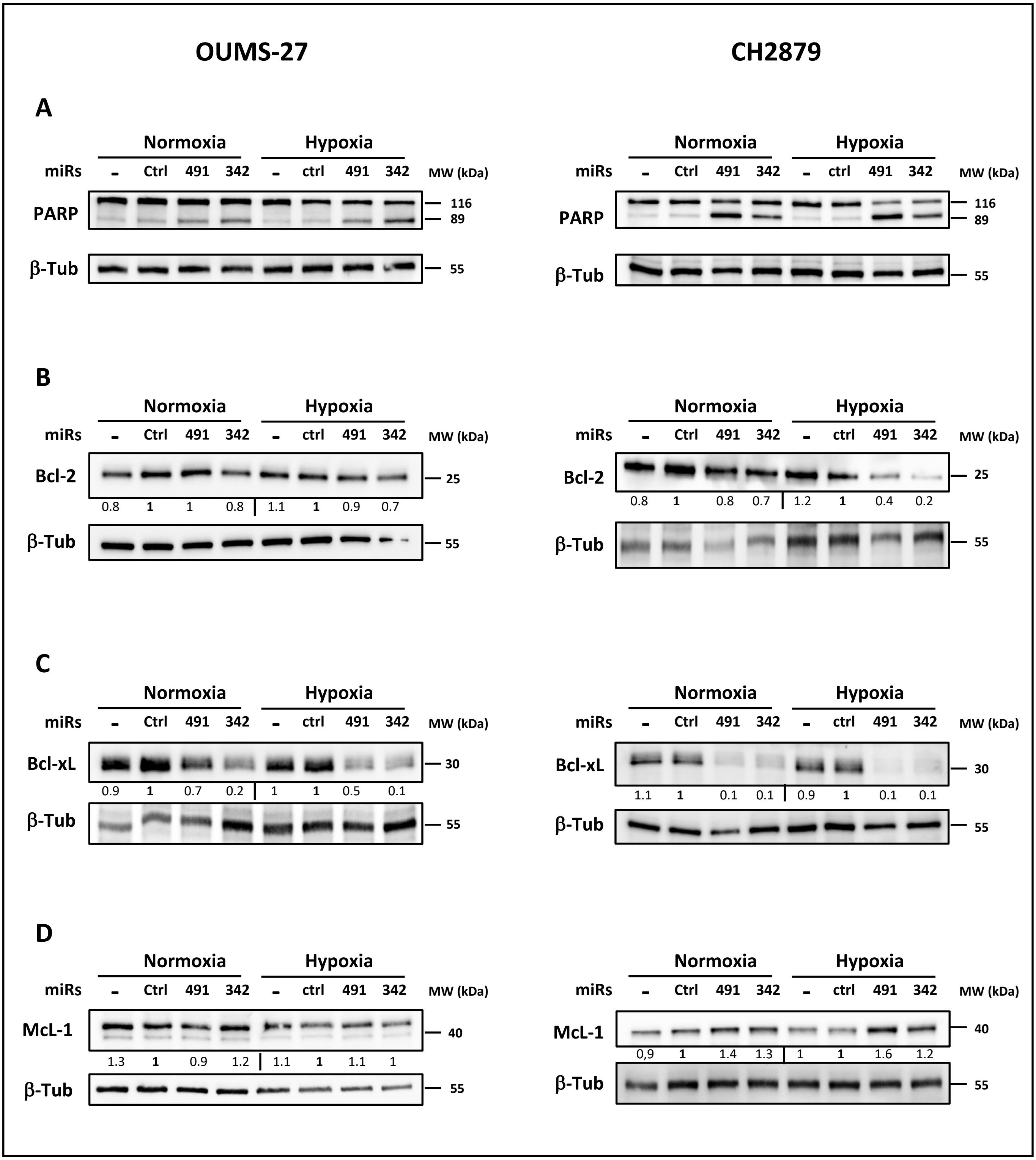

Supplementary Figure S7: Analysis of some apoptotic/antiapoptotic proteins in OUMS-27 and CH2879 cells. OUMS-27 cells (left panels) and CH2879 cells (right panels) were cultured and transfected as described in Figure 4. Analysis were carried out 72 h post-transfection. Protein extracts were analyzed with western blots to evaluate the levels of PARP (A), Bcl-2 (B), Bcl-xL (C), and McL-1 (D) versus β-tubulin (β-Tub). Representative blots of three independent experiments are shown. Protein expression levels are indicated at the bottom of the blots. They were normalized to β-tubulin, and to the corresponding miR-Ctrl for each oxic condition.

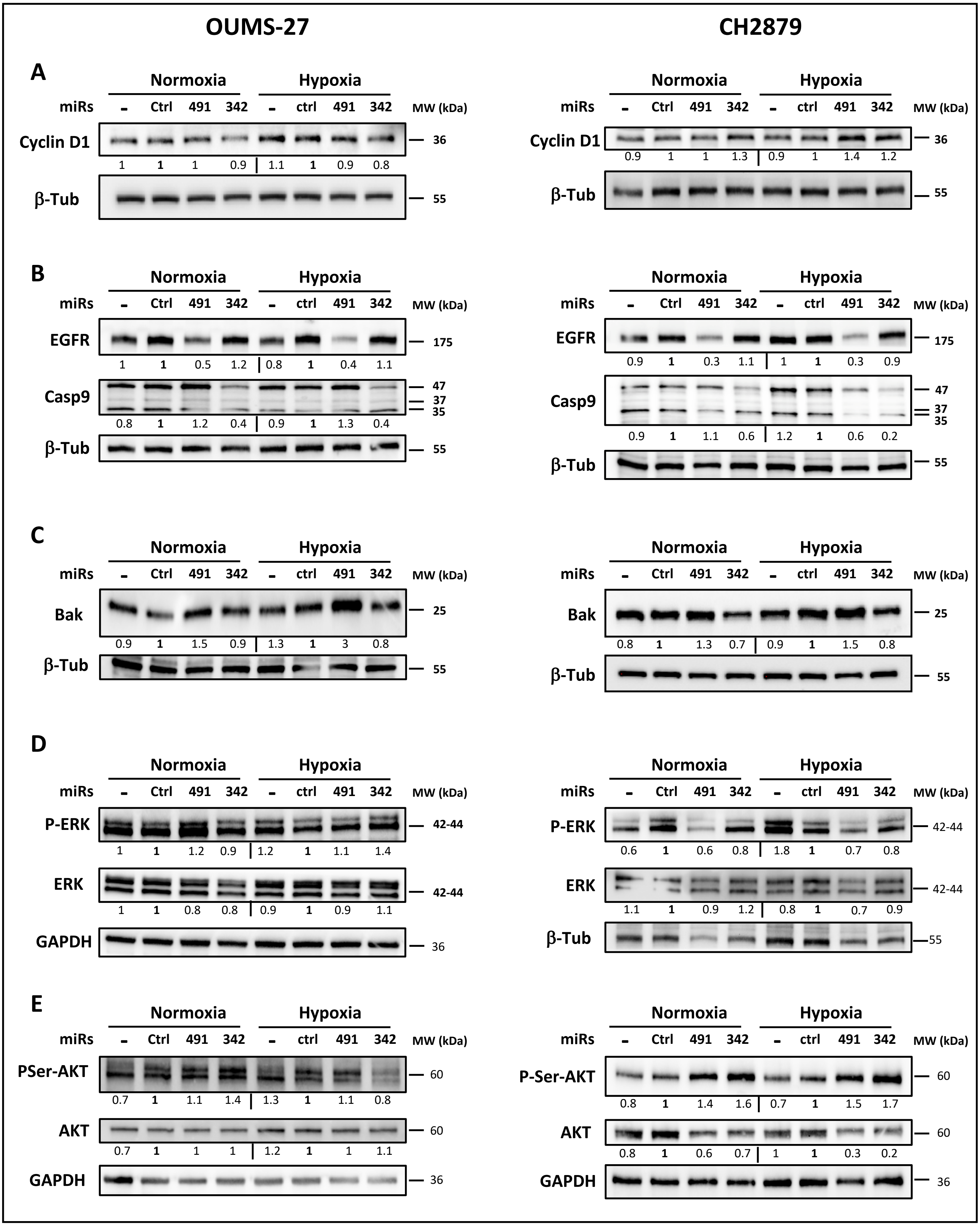

Supplementary Figure S8: Study of some survival/death proteins and mitogenic signaling pathways in OUMS-27 and CH2879 cells. OUMS-27 cells (left panels) and CH2879 cells (right panels) were cultured and transfected as described in Figure 4. Analysis were carried out 72 h post-transfection. Protein extracts were analyzed with western blots to evaluate the levels of Cyclin D1 (A), EGFR and Caspase-9 (B), Bak (C), P-Thr202/Tyr204-ERK, and ERK (D), P-Ser473-AKT, and AKT (E) versus β-tubulin (β-Tub) or GAPDH used as loading controls. Representative blots of three independent experiments are shown. Protein expressions are indicated at the bottom of the blots. They were normalized to β-tubulin or GAPDH, and to the corresponding miR-Ctrl for each oxic condition.

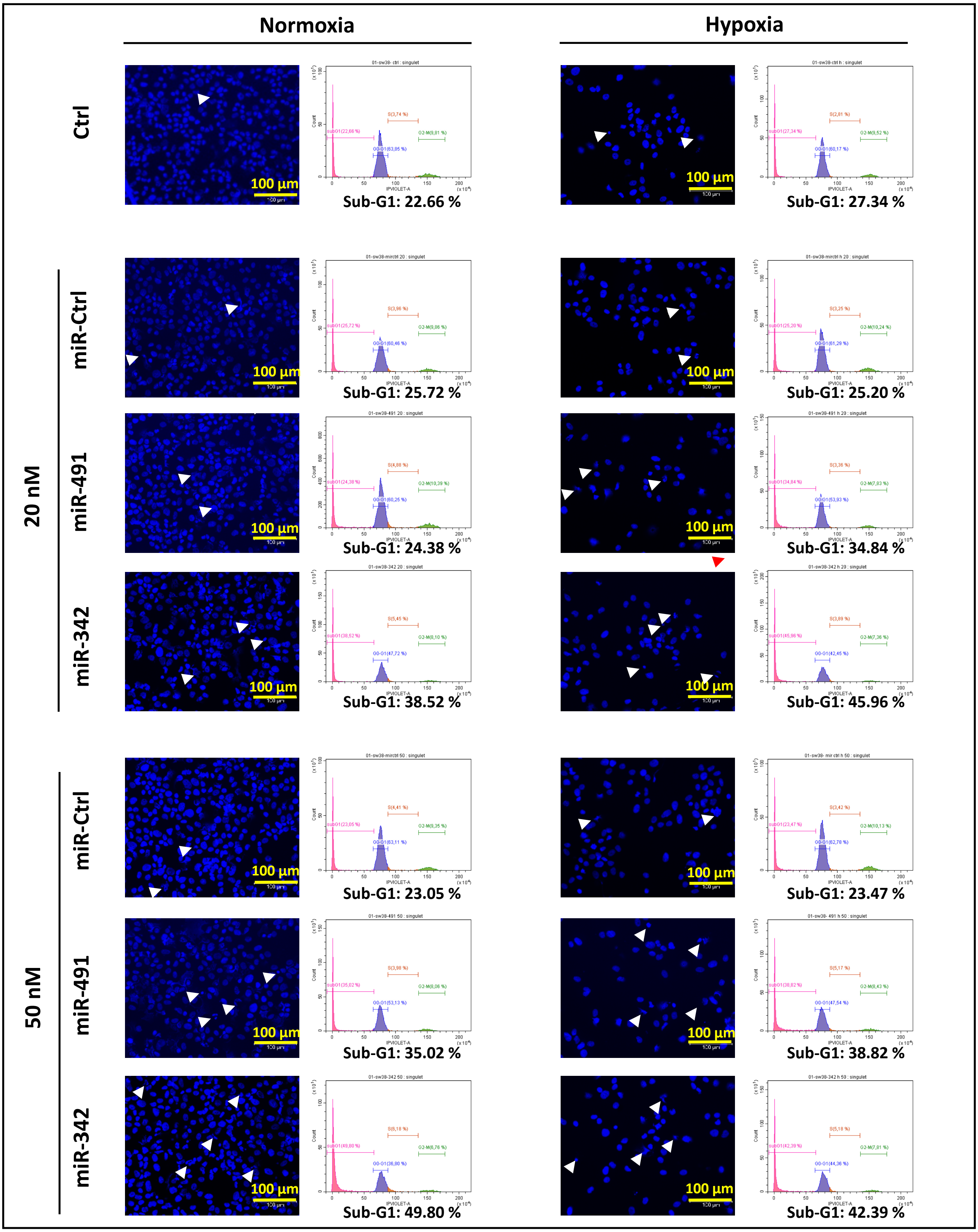

Supplementary Figure S9: Effects of miR-491-5p and miR-342-5p on SW1353 cells cultured in 3D. SW1353 cells were cultured in collagen sponge scaffolds under normoxia or hypoxia and transfected with miRNA as described in Figure 9. Analyses were carried out 72 h post-transfection. In the left part of each panel, nuclear morphology was obtained after DAPI staining. White arrowheads show cells with condensed and/or fragmented chromatin and cellular debris. In the right panels, DNA content histograms were obtained using flow cytometry. Images shown are representative of four independent experiments.

Table S1: **Predicted and direct targets of miR-491-5p.** MiRabel (http://bioinfo.univ-rouen.fr/mirabel/) uses data from the Pita, MiRanda, SVmicro and TargetScan prediction tools.

**Protein**	**Gene**	**miRabel score**	**Rank in PITA**	**Rank in miRanda**	**Rank in SVMicrO**	**Rank in TargetScan**	**Direct target**	**Citation**
Bcl-xL	***BCL2L1***	0.0018096199491993	1289	939	840	506	Validated	19-21
EGFR	***EGFR***	0.0078076198697090	582	1090	2932	2081	Validated	19, 22
ERK2 ou P42MAPK	***MAPK1***	0.0385214015841484	1291	2653	3725	3035	-	-
ERK1 ou P44MAPK	***MAPK3***	0.0440779998898506	4768	3214	393	2301	-	-
McL-1	***MCL1***	0.1036410033702850	1582	3940	4642	3957	-	-
Bak	***BAK1***	0.7485740184783936	5920	4397	10404	2779	-	-
Cyclin D1	***CCND1***	0.8208479881286621	7350	7322	2156	-	-	-
Caspase 9	***CASP9***	0.8376489877700806	-	2329	12290	3899	-	-
AKT	***AKT1***	0.9833989739418030	2502	-	9557	-	-	-

Table S2: Predicted and direct targets of miR-342-5p. MiRabel (http://bioinfo.univ-rouen.fr/mirabel/) uses data from the Pita, MiRanda, SVmicro and TargetScan prediction tools.

**Protein**	**Gene**	**miRabel score**	**Rank in PITA**	**Rank in miRanda**	**Rank in SVMicrO**	**Rank in TargetScan**	**Direct target**	**Citation**
Bcl-xL	***BCL2L1***	0.0015979099553078	274	333	694	1627	Validated	30
EGFR	***EGFR***	0.0031764199957252	266	1284	666	1933	-	-
Cyclin D1	***CCND1***	0.0166469998657703	2077	1721	1662	2148	Validated	30
ERK1 ou P44MAPK	***MAPK3***	0.0485971011221409	4858	3451	1078	1144	-	-
Bak	***BAK1***	0.0651884004473686	4660	4923	211	1649	-	-
Bcl-2	***BCL2***	0.1019570007920265	273	1676	5022	728	-	-
Caspase 9	***CASP9***	0.1040249988436699	5524	7	3686	3210	Validated	23
McL-1	***MCL1***	0.1049569994211197	2851	5648	754	3589	-	-
ERK2 ou P42MAPK	***MAPK1***	0.3654370009899139	692	-	3345	3785	-	-
AKT	***AKT1***	0.6705200076103210	6222	-	2309	3718	Validated	44
